# Understanding *Eimeria* infection for the treatment and prevention of chicken coccidian parasites

**DOI:** 10.3389/fcimb.2026.1756128

**Published:** 2026-03-27

**Authors:** Binh T. Nguyen, Rochelle A. Flores, Taesik Kim, Wongi Min

**Affiliations:** 1 College of Veterinary Medicine and Institute of Animal Medicine, Gyeongsang National University, Jinju, Republic of Korea; 2 Faculty of Animal Science and Veterinary Medicine, Thai Nguyen University of Agriculture and Forestry, Thai Nguyen, Vietnam; 3 Department of Rural and Biosystems Engineering, Chonnam National University, Gwangju, Republic of Korea; 4 Hoxbio, Business Center, Gyeongsang National University, Jinju, Republic of Korea

**Keywords:** coccidiosis, control strategies, *Eimeria*, poultry, prevalence and immunity

## Abstract

Globally, coccidiosis is one of the most economically important diseases affecting the poultry industry, and poultry plays a vital role in producing meat and eggs, providing high-quality, relatively inexpensive protein for humans. Due to its high feed efficiency compared to other industrial animals, this industry has gradually expanded worldwide, but its productivity is affected by various dangerous diseases. One of the pathogens is coccidiosis, caused by seven species of *Eimeria*, which infects the intestines and causes signs such as diarrhea, increased feed conversion rate, reduced egg production, and weight loss, and ultimately has a serious impact on the poultry farms. Anticoccidial drugs remain the first-line treatment for this parasitic disease in poultry farms to this day, and thus the emergence of antibiotic-resistant strains raises serious public health and environmental concerns. To reduce economic losses and avoid public health and environmental concerns, a better understanding of the epidemiology, infection mechanisms, host protective immunity, and control strategies of *Eimeria* species is necessary. The purpose of this review is to provide a comprehensive insight into chicken coccidiosis to effectively treat and prevent *Eimeria* infection. Effective control of coccidia can also improve the quality and productivity of chicken meat, which plays an important role in human food, and increase economic profits for chicken farms.

## 1 Introduction

The poultry industry has consistently been one of the world’s leading sources of animal protein (Fao, 2025). There are many pathogens of economic importance in the poultry farms (Muñoz-Gómez et al., 2025). One of the pathogens that compromises the efficiency of poultry mass production systems is coccidiosis, which can pose a threat to global food security and cause significant economic damage to the poultry industry (Mesa-Pineda et al., 2021; Lee et al., 2022). Coccidiosis is a major gastrointestinal disease of poultry, and the global cost of coccidiosis management is estimated at over £10.4 billion in 2016 prices and £12.9 billion in 2022 prices (Blake et al., 2020; Blake, 2025). The prophylactic use of various anticoccidial drugs in feed and water has long been a mainstream control strategy in modern poultry settings. Currently, the use of anticoccidial agents to control economic losses caused by coccidiosis in the poultry industry is significantly limited by public health and environmental concerns such as the emergence of drug-resistant parasites, possible drug residues in meat products, and soil and water contamination (Flores et al., 2022; Lee et al., 2022; Martins et al., 2022). Therefore, a variety of alternative strategies, including vaccines, biological agents, and natural products, have been considered to overcome concerns associated with the use of anticoccidial agents.

With the continued growth of the global population, protein demand is also increasing, and the poultry industry, with its relatively high feed efficiency compared to cattle and pigs, is gaining importance. Consequently, intensive farming systems are expanding and bird densities on farms are increasing, which is expected to further exacerbate the occurrence of poultry diseases including *Eimeria* infection (Martins et al., 2022). Additionally, chicken bedding waste is expected to increase the cost of raising chickens due to the price of processing bedding waste as well as environmental problems. Thus, litter waste is recycled multiple times to reduce the cost of raising chickens, which is thought to affect the incidence of coccidia depending on farm conditions such as humidity, temperature and litter management (Liu et al., 2014; Garcés-Gudiño et al., 2018).

The objective of this manuscript is to review the basic and general concepts of coccidiosis, including a detailed understanding of *Eimeria* infection, host immune responses, epidemiology, and control strategies. There is currently a global trend toward legally reducing the use of anticoccidial drugs in the poultry industry. This requires us to improve our understanding of the causative agent of coccidiosis and various control strategies for this harmful disease in order to achieve better control of *Eimeria* infection.

## 2 Literature search strategy

A systematic literature search was performed in PubMed, Scopus, and Google Scholar using combination of terms related to “coccidiosis” or “Eimeria infection” or “life cycle” or “prevalence” or “host immunity” or “control strategies” or resistance to anticoccidial drugs” “vaccines” or “immune-related genes” or “farm management” or “chickens” or “broilers” for publication dated as of September, 2025. Additionally, references in relevant articles and grey literature were manually searched to obtain eligible information. References were annotated only for papers that included at least an abstract written in English. Exclusion criteria comprised of non-original research (e.g., commentaries, editorials, conference abstracts), duplicate publication and publication with full text unavailable. Retrieved articles were imported into EndNote for further study analysis”.

## 3 Chicken coccidiosis

One of most economically important diseases in broilers is coccidiosis, induced by the causative agent *Eimeria* species belonging to the *Apicomplexa phylum* (Min et al., 2013; Mathis et al., 2025). These species are obligate intracellular parasites that infect gastrointestinal epithelial cells, which is the primary organ responsible for digestion and nutrient absorption (Nawarathne et al., 2021; Wickramasuriya et al., 2022). *Eimeria* infection destroys the intestinal epithelial cells and affects intestinal homeostasis, causing serious economic damage due to diarrhea, reduced feed utilization efficiency, reduced weight gain and egg production, and in severe cases, mortality. Beyond the destruction of epithelial cells, coccidiosis has also been demonstrated to disrupt the ecological balance of intestinal microbes thereby contributing to state of dysbiosis, the production of wetter excreta/litter conditions, and increased susceptibility to various secondary infections, including toxinogenic *Clostridium perfringens*, which causes necrotic enteritis (Dunlop et al., 2016; Macdonald et al., 2017; López-Osorio et al., 2020; Soutter et al., 2020; Campos et al., 2024, 2025).

There are seven species of *Eimeria* (*E. acervulina, E. maxima, E. tenella, E. mitis, E. praecox, E. brunetti*, and *E. necatrix*), which infect the intestines of chicken in a site-specific manner. Each *Eimeria* species infects a specific part of the intestine. For example, *E. acervulina* infects the duodenum, *E. maxima* invades the jejunum and ileum, *E. tenella* infects the cecum, *E. brunetti* targets the ileum and rectum, and *E. necatrix* localizes in the jejunum and ceca (López-Osorio et al., 2020; Lee et al., 2022; Britez et al., 2023). Along with the seven *Eimeria* species, new cryptic species known as operation taxonomic units (OUTs) OTUx, OTUy, and OTUz have been recently proposed as new species named *E. lata*, *E. nagambie* and *E. zaria*, respectively (Blake et al., 2021). These OTUs possess sufficient genetic and biological diversity to exhibit immunogenicity distinct from that of currently commercially available anticoccidial vaccines, and thus can be considered novel and distinct species (Blake et al., 2021; Britez et al., 2023). These new *Eimeria* species have been detected globally through molecular surveillance in Africa, Asia, South America, and Europe (Clark et al., 2016; Jaramillo-Ortiz et al., 2023), suggesting that the discovery of new species and their global detection will make research on host immunity and vaccine strategies to control *Eimeria* infection more complex than ever before.

Unsporulated oocysts are shed in the feces of infected chickens and survive in the environment for weeks to months, depending on temperature, moisture, oxygen availability, and litter conditions. These fresh oocysts consist of a thickened outer wall and a round mass with a nucleated zygote are not infectious, until the process of sporulation is initiated. Under optimal conditions (i.e. adequate temperature, oxygen and humidity), sporulation typically occurs within 1–2 days or within several days under suboptimal conditions. Once sporulated, oocysts can remain viable for extended periods, although their viability in litter may decline over time due to factors such as increased ammonia levels. Coccidiosis begins with the oral ingestion of sporulated/infectious oocysts that include four sporocysts, each with two sporozoites (López-Osorio et al., 2020; Ahmad et al., 2024). Once ingested by chicks, sporocysts from the sporulated oocyst will be released by the mechanical action of the gizzard. The digestive action of the bile and pancreatic fluids secreted into the duodenum destroys the outer wall of the sporocysts and releases the sporozoites into the lumen of the small intestine (Ahmad et al., 2024; Mathis et al., 2025). The sporozoite contains typical eukaryotic features and organelles, such as nucleus, mitochondrion, endoplasmic reticulum, and Golgi apparatus. It also has several features specific to the phylum, such as the conoid, apicoplast, micronemes and rhoptries (Burrell et al., 2020; Britez et al., 2023).

Free sporozoites invade epithelial cells in different regions of the gut depending on *Eimeria* species and undergo the life cycle of the coccidian parasite. After invasion, intracellular sporozoites develop into trophozoites, which then become larger and multiply asexually very quickly to form numerous merozoites known as merogony. Merozoites are released into the intestinal lumen after destroying the host’s originally infected intestinal epithelial cells. The merozoites then infect new, neighboring intestinal epithelial cells, completing the second merozoite cycle. This process can be repeated two or four times. After the asexual lifecycle, the resulting merozoites enter new intestinal epithelial cells and initiate gametogony or the sexual multiplication phase of the life cycle. During gametogony, the parasite develops into either microgamonts (male gametes) or macrogamonts (female gametes). Fertilization of male and female gametes results in the production of zygotes, which quickly form an oocyst wall and become an unsporulated oocyst that is excreted in the feces (Price, 2012; López-Osorio et al., 2020; Lee et al., 2022). All *Eimeria* species that infect chickens have a similar life cycle (Burrell et al., 2020; Rosłoń et al., 2025). The outer wall of the oocyst has a structure that is highly resistant to physical and chemical treatments, but is sensitive to freezing and high environmental temperatures. Sporulated oocysts can survive for long periods of time outside the host depending on environmental conditions (López-Osorio et al., 2020).

The clinical signs and pathogenicity of coccidiosis are influenced by multiple factors, including *Eimeria* species, chicken breed, infective dose, host-parasite interaction, composition of the host microbiota, feed ingredients, and the environmental conditions of poultry farms. Thus, the signs of *Eimeria* infection range from subclinical infection to death of the host (Mesa-Pineda et al., 2021; Martins et al., 2022). In commercial poultry production systems, exposure to pathogens is almost ubiquitous, and subclinical infections are prevalent (Karaer et al., 2012; Pajić et al., 2023). Conversely, the occurrence of overt clinical disease characterized by pathognomonic lesions, hemorrhagic enteritis and significant morbidity and mortality is more sporadic and contingent on the efficacy of management and control programs (Mesa-Pineda et al., 2021; Pajić et al., 2023; Ahmad et al., 2024). In general, different *Eimeria* species may show different clinical manifestations. *E. acervulina* and *E. mitis* cause fluid loss and decreased nutrient absorption, while *E. brunetti* and *E. maxima* can cause watery diarrhea, swelling of intestinal wall with petechiae and loosening of epithelium. *E*. *tenella* and *E. necatrix* cause complete destruction of intestinal epithelial cells, resulting in hemorrhages and mortality (Mesa-Pineda et al., 2021; Nawarathne et al., 2021). The predilection site and lesion scoring were described in **Table 1**. Feces from poultry farms typically contained three or four different *Eimeria* species (Morris et al., 2007; Flores et al., 2022; Lee et al., 2022), indicating that chickens can be simultaneously infected with multiple *Eimeria* species. Compared to infection with one species, multiple *Eimeria* infections cause more severe intestinal damage and increase mortality, and even make diagnosis in the field more difficult (Mesa-Pineda et al., 2021). Diagnosis of *Eimeria* infection involves counting the number of oocysts present in 1 gram of feces or poultry litter under a microscope or identifying the *Eimeria* species by polymerase chain reaction (PCR) by detecting the internal transcribed spacer (ITS) region of ribosomal DNA. This ITS region has been widely used in molecular diagnosis, phylogeny and taxonomy analysis (Haug et al., 2008; Kumar et al., 2015; Flores et al., 2022). To evaluate gross lesions caused by each *Eimeria* species, a lesion scoring technique, which is based on assigning a numeric lesion score from 0 (none) to 4 (most severe) has been commonly used both in the laboratory and field (Johnson and Reid, 1970; Mesa-Pineda et al., 2021; Nguyen et al., 2025).

**Table 1:** Predilection site and lesion score of Eimeria species on chickens.

*Eimeria* species	Prelilection sites	Pathogenicity	Lesions	Scoring system	Reference
*Eimeria acervulina*	Duodenum, jejunum	Medium	Whitish patches are observable on the serous surface of the wall, accompanied by hemorrhagic streaks. The signs include mucoid enteritis, transverse white plaques in the duodenum, a thickened intestinal wall, and characteristic ladder-like patterns.	0 to +4	Conway and McKenzie, 2007; López-Osorio et al., 2020
*Eimeria brunetti*	Caeca, rectum	High	Thin intestinal walls, mucoid with necrotic discharge, coagulation necrosis, along with mucoid bloody exudates in the lower intestine (rectum). Petechiae may be present on both the serosal and mucosal surfaces.	0 to +4	Conway and McKenzie, 2007; Nawarathne et al., 2021
*Eimeria maxima*	Jejunum, ileum	Medium	Distended intestine exhibiting hemorrhagic spots, mucoid exudate, and a thickened wall with blood-stained mucus. Petechiae, watery contents, a distinctive brownish-red mucosal surface, orange or bloody intestinal material, and ballooning, often accompanied by numerous petechiae in severe cases.	0 to +4	Conway and McKenzie, 2007; López-Osorio et al., 2020
*Eimeria mitis*	Lower jejunum, ileum	Low	No distinct lesions observed in the intestine; presence of mucoid exudates and slight thickening of the intestinal wall. In severe cases, the intestinal contents have a creamy appearance.	0 to +2	Conway and McKenzie, 2007; López-Osorio et al., 2020
*Eimeria necatrix*	Jejunum, ileum, caeca	High	Severe bleeding accompanied by a whitish mucoid discharge, with red spots visible on the intestinal wall. The intestine exhibits ballooning with white patches, petechiae, and mucoid, blood-filled exudates resembling a salt and pepper pattern. In cases of heavy infection, the intestinal length is typically reduced, and only unsporulated oocysts are observed in the caeca. The intestinal wall appears thickened.	0 to +4	Conway and McKenzie, 2007; Nawarathne et al., 2021
*Eimeria praecox*	Duodenum, jejunum	Low	There is no lesion; the intestinal surface of the duodenum appears slightly hemorrhagic with a mild mucous discharge, mucoid exudates, and whitish mucus indicative of heavy infection.	0 to +2	Conway and McKenzie, 2007; Nawarathne et al., 2021
*Eimeria tenella*	Caeca	High	Severe hemorrhaging with white and red spots observed on the intestinal wall, along with coarse clotted blood in the cecum lumen. The mucosa appears thickened and whitish. In cases of heavy infection, the average length of the ceca is reduced, and the cecal core is presented.	0 to +4	Conway and McKenzie, 2007; Nawarathne et al., 2021

## 4 Prevalence and distribution of coccidiosis

A recent review on 2024 (Badri et al., 2024) revealed that coccidiosis has a global prevalence of 44.3%. Notably, *E. tenella* was identified as the most common species, making up 38.7% of cases. Interestingly, the research suggests that humid subtropical climates, urban areas, and the autumn season are associated with higher rates of this disease. Another study in extensive backyard chickens in low-income and middle-income countries in 2024 claimed that coccidiosis had an estimated overall prevalence of 39%. Tropical climates generally had a higher percentage rate for coccidiosis (Muñoz-Gómez et al., 2024b). Regional prevalence estimates vary widely, ranging from 30.8% in the Southeast Asia, 66.0% in the Americas, 75.7% in the European region, to 80.5% in the Western Pacific region (Badri et al., 2024). There are seven commonly recognized *Eimeria* species that infect chickens; among them*, E. acervulina* and *E. tenella* are frequently identified as the most prevalent, with more than 80% prevalence (Lee et al., 2010a; Huang et al., 2017; Flores et al., 2022; Pakpinyo et al., 2025).

In the Americas, coccidiosis is highly prevalent, particularly in Brazil, Colombia, and the USA (Harat and Ansari, 2024). In a research paper on 250 farms in the southern region of Brazil, 96% were positive for *Eimeria*. *E. maxima* (63.7%), *E. acervulina* (63.3%), *E. tenella* (54.6%) were the most prevalent *Eimeria* species, and an average of 2.96 species were detected per farm (Moraes et al., 2015). Another study in free-range chicken farms from northeastern Brazil reported a prevalence of 59% (59/100) with varying species level distribution, where *E. necatrix* (25%) and *E. mitis* (18.3%) predominated, followed by lower frequencies for *E. mivati* (17.3%), *E. tenella* (12.4%), *E. brunetti* (9.9%), *E. acervulina* (9.1%), *E. praecox* (4.8%), and *E. maxima* (3.2%) (Silva et al., 2022). In Colombia, a nationwide survey of commercial broiler operations confirmed a farm-level prevalence of 92.8% (180/194 farms) with *E. acervulina* (35%), *E. tenella* (30.9%) and *E. maxima* (20.4%) as the predominant species. Mix infections were also frequent, and 41.1% (74/180) of *Eimeria* positive farms harbored at least 4 species (*E. acervulina* + *E. maxima + E. tenella +* others) (Mesa-Pineda et al., 2021). A recent study conducted in Ecuador identified the most prevalent combination of *Eimeria* species, which included *E. acervulina, E. maxima, E. necatrix*, and *E. praecox*, accounting for 23.9% of the findings (Cevallos-Gordon et al., 2024). Additionally, *E. tenella* was noted as a distinct species, representing 10.6% of the occurrences (Cevallos-Gordon et al., 2024). In Argentina and Chile, a study revealed an overall prevalence of *Eimeria* of 85.1% in family poultry production systems. Specifically, the prevalence was found to be 100% in Argentine broiler sheds, 73.7% in Argentine hen sheds, and 89.7% in Chilean hen sheds. Additionally, the study noted that mixed infections were common; however, *E. mitis* was detected in six out of eight single infections in egg production farms (Tomazic et al., 2025).

In Asia, the prevalence of poultry coccidiosis was comparatively lower than that observed in the Americas. In particular, it showed a higher detection rate of 55.96% in broilers, recognizing *E. acervulina* as the most common *Eimeria* species and showing that the greatest problem was in poultry farms in India (Harat and Ansari, 2024). Nepal reported a prevalence rate of coccidiosis at 1% (25/2271) compared to other diseases commonly occurring in poultry farms (Gompo et al., 2019). In Jammu and Kashmir, India, the prevalence of coccidiosis was recorded at 17.3% among broiler chickens and 12.4% among laying hens. Notably, the incidence of coccidiosis tended to be higher at altitudes below 4,000 feet (Harat and Ansari, 2024). Furthermore, a study of seasonal variations revealed that the prevalence of this disease was highest during the rainy season, with a rate of 32%. In contrast, the lowest prevalence was during the summer months, at 19.3%. Among the identified parasites, *E. tenella* exhibited the highest prevalence rate at 27.6%, followed by *E. necatrix* at 21.3% and *E. acervulina* at 16.5% (Harat and Ansari, 2024). A 5-year study in Beijing, Sichuan, Zhejiang, Shandong, Guangdong, Fujian, and Liaoning provinces, Inner Mongolia and Xinjiang Uygur autonomous regions in China showed an increase in the number of oocysts per gram and an increase in morbidity linked to non-prophylactic or low doses of coccidiostats (Zhang et al., 2013a). In Guangdong Province, the most commonly found species at the flock level were *E. acervulina*, *E. mitis*, *E. tenella*, and *E. necatrix*, with mixed infections observed in 93.25% of the farms surveyed and the highest morbidity and drug resistance rates (Liao et al., 2024). Coccidiosis in Korea remains highly prevalent in chicken farms. A recent large-scale epidemiological investigation survey reported an overall infection rate of 75% for 388 sampled farms (Flores et al., 2022). This study also showed that oocysts were detected in 84.5% of broiler farms, with native chickens accounting for 81.48%. Free-range chickens, which generally had an infection rate of 83.7%, showed a higher risk of *Eimeria* infection than cage-reared chickens (Flores et al., 2022). Furthermore, the most prevalent *Eimeria* species detected in positive samples were *E. acervulina* (98.6%), *E maxima* (84.8%) and *E. tenella* (82.7%), and were typically found as a mixed population averaging 4.2 *Eimeria* species per fecal sample (Flores et al., 2022). A recent study conducted in Thailand identified *E. tenella* as the most prevalent species in broiler farms, with a prevalence rate of 96%. This was followed by *E. acervulina* at 92% and *E. maxima* at 68%. Additionally, *E. brunetti* and *E. necatrix* were not detected in the surveyed populations (Pakpinyo et al., 2025).

Coccidiosis is notably widespread in Africa, with Ethiopia experiencing particularly high prevalence rates (Harat and Ansari, 2024). Using a binomial random effects model, the average prevalence across the Horn of Africa is estimated to be 0.21 (expected 21%). Ethiopia recorded the highest rate at 0.28 (28%), followed closely by the Republic of South Sudan and Kenya, both at 0.27 (27%) (Muñoz-Gómez et al., 2024a). In village chickens in Ethiopia, *E. praecox* was the most frequently detected species, occurring in 46% of single infections and 74% of co-infections (Luu et al., 2013). The predominant species identified in Bejaia Province in Algeria were *E. acervulina* (32%) and *E. tenella* (26.9%) (Debbou-Iouknane et al., 2018). All broiler farms in Medea Province were found to harbor five species of *Eimeria*. *E. mitis* was the most prevalent at 92.5%, followed by *E. tenella* at 81% and *E. necatrix* at 76% (Amina et al., 2025). Collectively, poultry housing systems are primarily categorized into cage and cage-free (non-cage) systems. In general, poultry housing system has a greater impact on the prevalence than the breed such as broiler or layer. Chickens raised in backyards or on cage-free systems had higher incidence of coccidiosis than caged chickens. Although prevalence and species profiles vary depending on the chicken breed, farm environment, climate, chicken age, detection period, region and country, *Eimeria* remains consistently entrenched across all surveyed continents, reaffirming the persistent global threat of coccidiosis. These results suggest that ongoing surveillance and regional-specific integrated control strategies are therefore essential to mitigate its continual impact on poultry health and productivity.

## 5 Host immunity to coccidiosis

*Eimeria* species is highly immunogenic in birds. The primary infection of coccidia can induce strong protective immune responses to the subsequent infection by the homologous parasite species. There is little or no cross-protection against subsequent challenge with a heterologous *Eimeria* species, and in some cases, even infection with a different strain of the same species is poorly protected, especially for *E. maxima* (Lee et al., 2010a; Min et al., 2013; Soutter et al., 2020; Jenkins et al., 2024). *Eimeria* species infecting intestinal intraepithelial cells stimulate both innate and adaptive immune responses, resulting in protective and long-lasting immunity. However, this primarily relies on cellular immunity, particularly mucosal immunity associated with the gut, rather than humoral immunity, which also plays a role in *Eimeria* infection (Min et al., 2013; Ahmad et al., 2016; Mesa-Pineda et al., 2021). There are also differences in immunogenicity between *Eimeria* species. In general, a relatively large number of oocysts are required to stimulate immunity against *Eimeria* species, with the exception of *E. maxima*, which is considered to be highly immunogenic because it induces a strong immune response with less than 100 oocysts (Chapman and Rayavarapu, 2007; Mesa-Pineda et al., 2021; Martins et al., 2022). Chicken breeds with genetic differences show different susceptibility to *Eimeria* infection. The SC (B^2^/B2) and TK (B^15^/B^21^) inbred chickens display different patterns of susceptibility to coccidiosis; SC is more resistant to the disease and TK is more susceptible (Choi et al., 1999; Yun et al., 2000b; Li et al., 2002). White Leghorn line C.B12 and 15I also showed different susceptibility to *E. maxima* infection: line C.B12 was more resistant to the disease and 15I was more susceptible (Bremner et al., 2021). Aviagen line A and B chickens have different responses to *Eimeria* infection, with line B chickens showing more susceptibility (Casterlow et al., 2011). The qualitative and quantitative aspects of the immune response may differ in embryos and newly hatched chickens compared to adult chickens (Alkie et al., 2019; Song et al., 2021). During the first two weeks after hatching, the number of intestinal lymphoid cells increases significantly, and the proportion of lymphocyte subtypes also changes with the age of the chicken (Bar-Shira and Friedman, 2006; Wickramasuriya et al., 2022). Development of Peyer’s patches in the intestine initiates during embryogenesis, and its compartmentalization and structural maturation take place after hatching (Zeinali et al., 2024), suggesting that age may be another factor influencing immune function. Additionally, egg yolk hyperimmune globulin (IgY) was collected from the eggs of hens repeatedly infected with *Eimeria* and fed to chickens. The chickens showed successful protection against subsequent challenges with *Eimeria* species (Lee et al., 2009b; Xu et al., 2013; Juárez-Estrada et al., 2021b). Taken together, these suggest that chicken breed, host age, maternal antibodies or infection dose may influence the host immune response to *Eimeria* infection.

### 5.1 Innate immunity

The innate immune system, also known as the nonspecific immune system, serves as the body’s first line of defense against invading pathogens and initiates rapid response mechanisms. Components of the innate immune barrier, consisting of physical, chemical, and microbial barriers, constitute the host’s most immediate form of immune defense (Wang et al., 2024a). Innate immune cells, including dendritic cells, granulocytes, macrophages, and natural killer (NK) cells, are widely distributed in various tissues and organs of the host animals, and they express pattern recognition receptors (PRRs) that recognize pathogens. Various mediator components such as complements, enzymes, chemokines and cytokines are also associated with innate immunity (Lu et al., 2023; Wlaźlak et al., 2023; Gomez-Osorio et al., 2023; Gao et al., 2024). In *Eimeria* infections, the intestinal mucosa functions as the first line of defense, with the gut microbiome residing on the mucosal surface. These commensal bacteria also produce antimicrobial substances, which enhance the host’s defenses (Min et al., 2004; Lee et al., 2022; Jebessa et al., 2025). Toll-like receptors (TLRs) play a critical role in the innate immune response of chickens by recognizing their specific ligands present on the *Eimeria* species and initiating immune responses (Chen et al., 2025a; Zhu et al., 2025).

Compared to the discovered chicken TLRs (Keestra et al., 2013; Rehman et al., 2021), very limited information exists on the TLR ligands of chicken *Eimeria* species. One of the TLR ligands is profilin (known as 3-1E, a 19-kDa soluble protein), which is expressed in the developmental stages of *E. tenella* and is conserved across *Eimeria* species (Fetterer et al., 2004; Kim et al., 2019). Transgenic *E. tenella* expressing profilin showed enhanced protective immunity against challenge infection, with increased antigen-specific cell-mediated immunity and interferon gamma (IFN-γ)-secreting lymphocytes (Tang et al., 2018). Although there is no information on the ligand for profilin in chickens, profilin of *Toxoplasma gondii* is recognized by TLR11 and TLR12 in mice (Koblansky et al., 2013; Hatai et al., 2016). Aspartyl protease was recently identified in *E. tenella* as a potential chicken TLR15 ligand. Chicken macrophage HD11 cells or dendritic cells treated with aspartyl protease stimulated the production of proinflammatory cytokines and chemokines (IL-1β, IL-6, CXCLi1, CXCLi2), pathogen-killing mediators such as nitric oxide and reactive oxygen species, and TLR15 (Chen et al., 2025a). Similarly, macrophage HD11 cells transfected with *E. tenella* sporozoites and *E. praecox*-infected chickens showed increased expression level of TLR15 (Sumners et al., 2011; Sandholt et al., 2021b). Chickens exhibited highly dynamic expression profiles of TLRs over time and in different tissues following *E. tenella* infection (Wang et al., 2024b; Zhu et al., 2025). Interestingly, polymorphic variation in the sequence of TLRs may influence the recognition of their ligands and modify the innate immune response of the host against pathogenic invasions, including *Eimeria* species (Rehman et al., 2021). To date, information on the direct evidence between chicken TLRs and their corresponding ligands in Eimeria species is very limited, and therefore, more extensive studies are needed to understand and enhance innate immunity against Eimeria infections.

Innate humoral components such as CpG oligodeoxynucleotides (CpG-ODNs) and antimicrobial peptides (AMPs) are also another mechanism that helps eliminate invading pathogens following binding to their receptors or ligands (Wlaźlak et al., 2023; Wang et al., 2024a). CpG-ODN increased resistance to *E. acervulina* infection in a normally susceptible chicken breed (TK), but not in the more disease-resistant SC breed. *In ovo* administration of CpG-ODN also improved protective effects against *Eimeria* infection (Dalloul et al., 2004; Dalloul and Lillehoj, 2005). The anticoccidial roles of AMPs such as cathelicidin (CATH), NK-lysin, and liver-expressed antimicrobial peptides (LEAP) have been studied. Chicken NK-lysin is highly upregulated in intestinal intraepithelial lymphocytes (IELs) of *Eimeria*-infected chickens (Min et al., 2005; Kim et al., 2021). NK-lysin-treated chickens showed promising effects in broilers infected with *E. acervulina* (Wickramasuriya et al., 2021). In general, LEAP2 was downregulated in *E. maxima*-infected chickens (Casterlow et al., 2011; Sumners et al., 2011; Su et al., 2018). CATH3 expression was downregulated in the jejunum of *E. praecox*-infected chickens on day 3 post-infection (Sumners et al., 2011; Wang et al., 2020), whereas CATH2 and CATH3 were upregulated in the cecum of *E. tenella*-infected chickens at 3 hours post-infection (Wang et al., 2024b). Collectively, chickens generally showed highly variable AMP expression patterns over time and in different tissues after *Eimeria* infection.

The involvement of cellular components such as dendritic cells (DC), granulocytes, macrophages and NK cells in innate immunity to chicken coccidiosis has been documented. Chickens immunized with exosomes obtained from *Eimeria* antigen-loaded dendritic cells or with exosomes derived from the serum of *E. tenella*-infected chickens exhibited protective immunity against subsequent *Eimeria* challenges (Del Cacho et al., 2011; 2012; Del Cacho et al., 2016). *E. tenella* 14-kDa phosphohistidine phosphatase stimulated maturation of chicken DC and triggered DC-induced T cell immunity. Additionally, treatment of DC isolated from chicken spleen tissue with *E. acervulina* glyceraldehyde-3-phosphate dehydrogenase enhanced Th1-type immune responses (Lakho et al., 2020, 2022). Chicken splenic macrophages and monocyte-derived macrophages stimulated with *E. tenella* sporozoites produce nitric oxide (NO), an important mediator of innate immunity (Lillehoj and Li, 2004) and reduce the susceptibility to the invasion of *Toxoplasma gondii*, respectively (Zhang et al., 2023b). NK cells are found in higher densities in the intestine and are particularly abundant in IELs of young chickens (Alkie et al., 2019). NK cells can induce target cell lysis by secreting substances such as perforin and granzymes or influence apoptosis through Fas ligand pathways (Wang et al., 2024a). Expression of perforin and granzyme A were increased in cecal tissues of *E. tenella*-infected chickens (Wattrang et al., 2016). Increased expression of NK-lysin was observed in both IELs stimulated with *E. necatrix* sporozoites and IELs from chickens infected with *E. necatrix*. Full-length chicken NK-lysin or NK-lysin peptides exhibited anticoccidial activity both *in vitro* and *in vivo* (Lee et al., 2013; Kim et al., 2017, 2021; Wickramasuriya et al., 2021).

Heterophil extracellular traps (HETs) are an innate immune defense mechanism in birds, particularly chickens, analogous to mammalian neutrophil extracellular traps. HETs consist of DNA, histones, and elastase, released by heterophils to trap and kill invading microorganisms or their secondary metabolites (Chuammitri et al., 2009; Chen et al., 2022b; Jiang et al., 2022). Interestingly, HETs were induced by chicken heterophil cells stimulated with *E. tenella* sporozoites (Wei et al., 2019; Rentería-Solís et al., 2024). Eosinophils primarily function as immune cells that protect against parasites and are known to release cytotoxic proteins to kill parasites (Wang et al., 2024a). Increased eosinophilic infiltration was observed in the jejunum of *E. necatrix*-infected chickens (Chen et al., 2025c). Goblet cells produce mucus composed of glycoproteins that covers almost the entire gastrointestinal epithelium and acts as a physical barrier against invasion by pathogens, including *Eimeria* species (Lee et al., 2022). *Eimeria* infection crosses the mucus interface, thus affecting goblet cell numbers and mucin secretion, which may influence the ability of other pathogens to invade the gut (Chen et al., 2025b). Chicken mucin purified from the duodenum and cecum inhibited the invasion of *E. tenella* sporozoites into Madin-Darby bovine kidney (MDBK) cells, but parasite invasion was not affected by bovine mucin (Tierney et al., 2007). It is worth to noting that broiler breeders with efficient innate immunity had higher resistance to *Eimeria* infection (Swaggerty et al., 2011). In brief, various cellular components of the innate immune response significantly influence resistance to *Eimeria* infection, contributing to protective immunity, and chickens with stronger innate immunity were generally shown to have greater resistance to coccidiosis.

### 5.2 Adaptive immunity

Innate and adaptive immune responses are both important in the prevention of chicken coccidiosis. The adaptive immune response, also known as acquired immunity, consists of highly specific T and B lymphocytes and is regulated by antigen-specific receptors expressed on the surface of these lymphocytes (Min et al., 2013; Wang et al., 2024a). This immunity is essential for providing long-lasting protection against *Eimeria* infection in chickens through immunological memory. *Eimeria* species produce a variety of antigens during its complex intestinal life cycle after infection, making it difficult to accurately and thoroughly understand humoral and cell-mediated immunity. Although studies on immunoglobulins have shown that they play a considerable role in controlling infections by *Eimeria* species, protective immunity primarily relies on T cells (Soutter et al., 2020). In the *Eimeria* life cycle, the asexual stages including sporozoite and merozoite are generally considered more important targets of protective immunity against subsequent infections than the sexual stages (Min et al., 2013; Britez et al., 2023; Gao et al., 2024; Jebessa et al., 2025).

Because *Eimeria* species infect chickens by fecal-oral route and multiply in the intestinal epithelium, *Eimeria*-specific immunoglobulins are produced during *Eimeria* infection in association with gut-associated lymphoid tissue (GALT), which contains more than 70% of the body’s immune cells (Wickramasuriya et al., 2022; Chen et al., 2025b). Chickens infected with *Eimeria* species produce large amounts of parasite-specific IgA, IgM, and IgG antibodies, which are present in serum, bile, and intestinal secretions (Pierce et al., 1962; Trees et al., 1989). Antibody titers peaked 2–3 weeks after *Eimeria* infection and then declined, showing highly dynamic patterns of antibody levels depending on birds. There were also differences in the antigenicity of antibody production depending on *Eimeria* species (Rose and Long, 1962; Smith et al., 1994b; Wallach, 2010). While *Eimeria* exposure consistently induces a robust antibody response, humoral immunity does not consistently correlate with clinical protection, as resistance to coccidiosis is predominantly cell-mediated (Wallach, 2010; Soutter et al., 2020). For instance, when two chicken breeds with different disease susceptibility to coccidiosis were infected with *E. acervulina*, *E. maxima* and *E. tenella*, the titers of serum IgM and IgG, and biliary IgA were similar between the two breeds, suggesting a lack of correlation between antibody titers and disease susceptibility (Lillehoj and Ruff, 1987). Bursa-less chickens with significantly reduced or undetectable serum antibodies were resistant to *E. tenella* infection, raising questions about the role of humoral immune responses in protective immunity against coccidiosis (Pierce and Long, 1965). Chickens that received immune serum or γ-globulin collected from *E. tenella* infections by intravenous or intraperitoneal injection did not exhibit protective immunity against subsequent challenge infections, indicating that passive immunity did not function properly (Pierce et al., 1963). On the other hand, after offspring chicks of *E. maxima*-infected hens were infected with the homologous *Eimeria* species, the anticoccidial effect of maternally transferred antibodies was evaluated by fecal oocyst production. A good correlation was found between parasite-specific antibody titers in sera or egg yolks and resistance to the homologous *Eimeria* species (Smith et al., 1994a, b).

There are numerous studies on the roles of antibodies generated by whole live parasites in coccidia defense. Cecal contents from immunized chickens contained high levels of IgA, whereas IgG and IgM were barely detectable or absent. In cecal mucosa of chickens infected with *E. tenella* parasite-specific IgM, IgA and IgG were detected (Davis et al., 1978; Girard et al., 1997). Sera and cecal contents from chickens infected with *E. tenella* inhibited sporozoite invasion activity in a chick kidney cells (Davis et al., 1978; Davis and Porter, 1979). Anti-sporozoite IgA antibodies in gall bladder bile of *E. tenella*-infected chickens influence reduction in the number of sporozoites in the intestinal lumen during subsequent reinfection (Rose and Hesketh, 1987; Yun et al., 2000a). Diets supplemented with hyperimmune egg yolk IgY powder produced from hens immunized with mixed live oocysts of *E. acervulina*, *E. maxima*, and *E. tenella* provided protective immunity against infection with all three *Eimeria* species (Lee et al., 2009a; b; Juárez-Estrada et al., 2021b). In another study, yolk IgY antibodies raised by immunizing hens with five *Eimeria* species, including *E. tenella*, were protective against *E. tenella* challenge infection (Xu et al., 2013; Gadde et al., 2015). Chicks hatched from eggs collected between days 28 and 39 after *E. maxima* infection were challenged with either homologous or heterologous *Eimeria* species. The protective effects of maternally transferred antibodies were demonstrated by an 82-87% reduction of fecal oocyst production in *E. maxima* challenge and 43-62% reduction of fecal oocyst production in *E. tenella* challenge, indicating partial protection against heterologous *Eimeria* species (Smith et al., 1994c). In addition to studies on the effects of antibodies produced against the whole parasite, there is also research on the anticoccidial role of monoclonal and polyclonal antibodies produced for parasite-specific antigens. Chicken monoclonal antibody (6D-12-G10), which detects the apical complex of *E. acervulina* sporozoites inhibited the invasion of sporozoites into chicken CD8 T cells *in vitro* (Sasai et al., 1996; Min et al., 2001). Monoclonal antibodies to *E. tenella* sporozoites lysed the parasite in the presence of complement and protected chickens against challenge infection with homologous *Eimeria* species (Crane et al., 1988). Furthermore, chickens treated intraperitoneally with a mouse monoclonal antibody against *E. tenella* gametocyte antigen 56 had approximately 70-78% reduced fecal oocyst shedding in the homologous challenge (Wiedmer et al., 2017). Chicks hatched from eggs of hens immunized subcutaneously with four recombinant antigens exhibited protective effects against mixed infection with *E. acervulina*, *E. maxima*, and *E. tenella* (Yang et al., 2022). Offspring chicks of hens immunized orally with sonicated gametocyte proteins of *E. tenella* showed reduced fecal oocyst shedding against homologous challenge (Hafeez et al., 2007). *Eimeria* infection in chickens stimulates production of parasite-specific antibodies (IgA, IgM, and IgG) in association with gut-associated lymphoid tissue and systemic immune responses. Antibody levels peak 2–3 weeks after infection and vary by individual birds. The role of antibodies in protection against *Eimeria* infection may vary depending on the circumstances, and their exact effect is currently still controversial. Although opinions differ regarding the role of antibodies in the protective immunity of chickens infected with *Eimeria*, and further research is needed, it is highly likely that antibodies play some role. Generally, T cell-mediated cellular immunity is considered to play a more important role than B cell-mediated immunity in *Eimeria*-infected chickens.

T cell-mediated immune responses are critical for the defense against intracellular pathogens, especially *Eimeria* species, and are modulated by a combination of various cytokines and subsets of lymphocytes (Lee et al., 2022; Wang et al., 2024a). The major T cell subsets are CD8^+^ cytotoxic lymphocytes (CTLs) and CD4^+^ T helper (Th) cells, both of which are associated with protective immunity against *Eimeria* infection. CD4^+^ T cells differentiate into Th1, Th2, Th17 and Tregs, each generating different cytokines and playing distinct roles in immune responses. CD8^+^ T cells secrete perforin and granzymes, directly killing infected cells and inducing apoptosis in target cells (Min et al., 2013; Soutter et al., 2020; Wang et al., 2024a).

Partially thymectomized birds had less resistance during primary *E. tenella* infection compared to normal birds (Pierce and Long, 1965). Chickens treated with cyclosporin A, an immunosuppressant that inhibits T cell activation, were shown to lose protective immunity at the time of secondary infection with *E. tenella* (Lillehoj, 1987). In contrast, turkey *Eimeria* does not infect chickens, but chickens treated with cyclosporin A permitted the complete intracellular development of turkey *Eimeria* (Kogut and Eirmann, 1991). Cell-mediated immune responses, such as delayed hypersensitivity and leukocyte stimulation, to oocyst antigen extracts were observed in chickens immunized with a commercial coccidiosis vaccine (Giambrone et al., 1980). Naive chickens administered intraperitoneally with transfer factor prepared from splenic lymphocytes of chickens immunized with a commercial coccidiosis vaccine showed delayed hypersensitivity to oocyst antigen and protective immunity against *E. tenella* challenge infection (Klesius and Giambrone, 1984). Selective depletion of T lymphocytes by intraperitoneal inoculation of CD4 and CD8 mAbs revealed that CD4^+^ lymphocytes play a significant role in controlling primary infection with *E. tenella*, but not with *E. acervulina*. CD8^+^ lymphocytes appeared to play a role in resistance to secondary infections with *E. tenella* and *E. acervulina*. CD8^+^ lymphocytes contained the most sporozoites in duodenal tissues of chickens infected with *E. acervulina*, whereas sporozoites were occasionally found in CD4^+^ lymphocytes, but not in B lymphocytes (Trout and Lillehoj, 1995; 1996). In chickens infected with *E. maxima*, CD3^+^, CD4^+^, and CD8^+^ lymphocytes increased in IELs during the primary infection, whereas only CD4^+^ T cells increased after secondary infection (Hong et al., 2006b). In peripheral blood leukocyte T lymphocytes of *E. tenella*-infected chickens, CD8^+^ cells increased 8 days after the primary infection, while CD4^+^ cells decreased at days 9-10, but no changes were observed after the secondary infection (Breed et al., 1996). Sonicated *E. tenella* sporozoite antigen stimulated spleen cells in *E. tenella*-infected chickens and induced proliferation of the TCRαβ^+^ population, but not the TCRγδ^+^ population. The proliferated T cells were CD4^+^CD8^-^, CD4^+^CD8αα^+^, and CD4^-^CD8 αβ^+^, and CD4^+^CD8αα^+^ T cells generally had the highest response (Wattrang et al., 2019, 2023). Additionally, CD4^+^ and CD8^+^ subpopulations were increased in the splenic lymphocytes or peripheral blood mononuclear cells (PBMC) of chickens immunized intramuscularly with *Eimeria* common antigen 14-3-3 (Liu et al., 2018), *E. maxima* antigen (EmARM-β) (Chen et al., 2022a), and a multiepitope antigen (*E. necatrix* NA4 antigen, *E. tenella* surface antigen 1, *E. acervulina* lactate dehydrogenase and *E. maxima* calmodulin-domain protein kinase) (Yu et al., 2022). Collectively, cell-mediated immunity (CMI), particularly intestinal mucosal immunity, plays a more important role than humoral immunity in *Eimeria*-infected chickens. Because *Eimeria* species infect in a region-specific manner and induce species-specific immunity, understanding and studying the function of lymphocytes in infected intestinal tissue is challenging. Therefore, limited research has been published to date, resulting in a paucity of information. Extensive research is needed to understand the role of CMI in coccidiosis-infected chickens.

### 5.3 Cytokines and immune-related genes

Innate immune response plays a key role in the initiation of adaptive immunity. Complex cytokine signaling mediates the interaction between innate and adaptive immunity to coordinate the protective immune responses against pathogens (Alkie et al., 2019; Lu et al., 2023; Wang et al., 2024a). In inbred strains SC and TK, which differ in susceptibility to *E. acervulina* infection, IFN-γ transcript expression levels were higher in SC chickens than in TK chickens in cecal tonsils and splenic lymphocytes, whereas reduced expression of IFN-γ was observed in duodenal IELs in both inbred strains. TGF-β mRNA levels were increased in lymphocytes obtained from cecal tonsil, spleen, and duodenum (Choi et al., 1999). White Leghorn line C.B12 (resistant line) infected with *E. maxima* showed earlier expression of IFN-γ and IL-10 transcripts in the jejunum tissue than 15I (susceptible line), although these two cytokines were increased in both chicken breeds (Bremner et al., 2021). Transcript levels of Th1-related cytokines IFN-γ, IL-2, IL-10, IL-12, IL-15, IL-16, and IL-18 were increased in duodenum IELs of chickens primarily infected with *E. acervulina*. Transcript levels of IFN-γ, IL-10, and IL-12 were increased in cecal IELs of chickens infected primarily with *E. tenella*, while the expression of IL-15, IL-16, and IL-18 was unchanged and the expression of IL-12 was decreased. Transcripts for Th2-related cytokines IL-13 and GM-CSF were upregulated after the primary or secondary infection with both *Eimeria* species, while IL-4 and IL-13 mRNA levels were decreased (Hong et al., 2006a). There was no difference in IL-2 production in serum, spleen, duodenum, and cecum between SC and TK strains in the primary *E. tenella* infection, but SC chickens showed higher IL-2 levels in serum and duodenum than TK chickens in the secondary infection (Li et al., 2002; Lillehoj et al., 2004).The cecum of *E. tenella*-infected chickens, similar to the jejunum of *E. maxima* infected-chickens, induced increased IL-1β, IFN-γ, and CC chemokines K203 and macrophage inflammatory factor 1β (Laurent et al., 2001). Additionally, in the cecal tissues of chickens infected with a commercial vaccine including *E. acervulina*, *E. maxima* and *E. tenella*, transcript levels of cytokines (IL-2, IL-10, TGF-β, IFN-γ) and T lymphocytes markers (CD4, CD8) were generally unchanged or increased. IL-1β was downregulated on day 7 post-infection and upregulated on day 14 post-infection. IL-8 was downregulated at all time points on days 7, 14 and 21 after infection (Lima et al., 2025). Cecal tissues of *E. tenella*-infected chickens showed upregulated mRNA expression of Th1 cytokines (IFN-γ, IL-2, IL-12), Th17-related cytokines (IL-17A, IL-17F, IL-22), and Treg-related cytokines (IL-10, TGF-β, CTLA) (Yu et al., 2021). In the duodenum of chickens infected with *E. acervulina*, IL-6 and IL-18 transcript levels were downregulated, IL-10 was upregulated, and IL-18 and IL-22 remained generally unchanged. The expression levels of IL-6, IL-8, IL-10, IL-18 and IL-22 were increased in the cecum of *E. tenella*-infected chickens (Zhou et al., 2023). Interestingly, upregulated expression levels of IL-6, IL-12, IL-17, IFN-γ, and IL-4 were detected in the early stage of infection in the bursa of chickens infected with *E. tenella*, whereas IL-1β was upregulated in the late stage of infection (Zhu et al., 2025). To better understand the function of cytokines in *Eimeria* infection, several studies have used antibodies against cytokines, one of which is IL-10, an anti-inflammatory cytokine with the ability to downregulate proinflammatory cytokines. Diets supplemented with egg yolk powder produced from hens immunized with IL-10 peptides were protective against weight loss induced by *Eimeria* challenge, but had no effect on reducing oocyst production in feces (Sand et al., 2016). Similarly, diets supplemented with dried egg products containing IL-10 or IL-4 antibodies had weak anticoccidial effects (Arendt et al., 2019; Abdul Rasheed et al., 2020; Fries-Craft et al., 2023). Overall, resistant chicken breeds induce higher levels or earlier expression of IFN-γ, in response to *Eimeria* infection compared to susceptible breeds. However, further research is needed to understand the precise role of other cytokines in *Eimeria*-infected chickens.

Among the IL-17 family cytokines, chicken IL-17A was first identified from the cDNA library of duodenal IELs derived from *E. acervulina*-infected chickens, followed by cloning of IL-17F and IL-17D (Min and Lillehoj, 2002; Kim et al., 2014; Diaz et al., 2016; Lu et al., 2023). IL-17F and IL-17A were upregulated in the cecum and jejunum of chickens infected with *E. tenella* and *E. maxima*, respectively. IL-17 receptor A (IL-17RA), which acts as a receptor for IL-17A and IL-17F, was either unchanged or downregulated (Kim et al., 2014). In cecal IELs of chickens infected with *E. tenella*, transcript levels of IL-1β, IL-6, and IL-17A were upregulated during early infection and then decreased thereafter, whereas TGF-β was upregulated until day 3 post-infection (Zhang et al., 2013b). Intravenous administration of IL-17A antibodies to infected chickens to neutralize IL-17A decreased IL-17A, IL-6, and TGF-β in both PBMCs and cecal IELs, but increased IL-2 and IFN-γ expression, resulting in a decrease in immunopathological findings (Zhang et al., 2013b). Similarly, treatment of IL-17A antibody to *E. tenella*-infected chickens reduced the maturation of schizonts, and also decreased cecal lesions (Del Cacho et al., 2014). Contrary results were observed in the recombinant IL-17A-treated and infected groups with increased fecal oocyst production, exacerbated lesion score and reduced body weight gain (Zhang et al., 2013b). Chickens infected with transgenic *E. necatrix* expressing IL-17A produced more fecal oocyst, which is thought to negatively affect immunogenicity against *Eimeria* infection without affecting an increase in intestinal lesion scores (Duan et al., 2024). Among the Th17 family cytokines, IL-17A is the most studied cytokine in *Eimeria*-infected chickens and is thought to have a negative function in coccidiosis, although this is based on limited studies.

Transgenic *Eimeria* expressing cytokines such as IL-2, IL-4 and IL-1β has been constructed through stable or transient transfection to increase or modulate host immunity against *Eimeria* infection (Li et al., 2015; Fatoba and Adeleke, 2020; Duan et al., 2024). Chickens infected with transgenic *E. mitis* expressing IL-2 exhibited improved cellular immunogenicity, with increased amounts of *E. mitis*-specific IFN-γ-secreting lymphocytes in PBMCs, and also showed reduced fecal oocyst shedding in both the primary and secondary infections (Li et al., 2015; Khan et al., 2016). Chickens inoculated with transgenic *E. mitis* expressing IL-4 shed fewer oocysts with impaired reproductive potential, although studies are needed to determine whether this effect occurs directly or indirectly (Khan et al., 2016). Compared to wild-type *E. necatrix*, transgenic *E. necartix* expressing IL-1β showed similar fecal oocyst production, but enhanced immunogenicity with further reduced fecal oocyst production after the secondary immunization and challenge infection (Duan et al., 2024).

Recently, transcripts obtained through RNA sequencing can be classified into pathways or specific domains with transcriptome analysis tools such as the Kyoto Encyclopedia of Genes and Genomes (KEGG) and Gene Ontology (GO), which helps to identify general trends in expressed transcripts. In the bursa of chickens infected with a commercial coccidia vaccine, tumor necrosis factor receptor super family 6 B (TNFRSF6B) showed the highest abundance in cytokine-cytokine receptor interaction pathway. Overexpression of TNFRSF6B upregulated transcript levels of IL-1β, IL-2, IL-6, IFN-γ and TNF-α, suggesting that TNFRSF6B is involved in the host immune response to coccidiosis (Guo et al., 2020). Duodenal tissues from chickens infected with *E. acervulina* induced an increase in the number of immune-related genes on day 6 post-infection compared to day 4 post-infection (Nguyen et al., 2025). Duodenal tissues infected with precocious strain of *E. acervulina* showed mRNA expression of IFN-γ, interferon regulatory factor 1, and IL-10 as early as 6 hours after infection (Zhang et al., 2024). Differential expression genes (DEGs) that were detected on days 4 and 7 post-infection in chicken with *E. maxima* were identified in the jejunum (SLC7A5, IL1R2, GLDC, ITGB6, ADAMTS4, IL1RAP, TNFRSF11B, IMPG2, WNT9A, and FOXF1) and in the cecum (FSTL3, RBP7, CCL20, DPP4, PRKG2, TFPI2, and CDKN1A), indicating that the expression profiles of immune-related DEGs differed between jejunum and cecum due to site-specific infection of *E. maxima* (Jebessa et al., 2024). In cecal tissues of *E. tenella*-infected chickens, immune activation was observed from day 3, and the highest number of immune-related DEGs was observed on day 10 post-infection. DEGs upregulated on day 3 or 4 were IFN-γ, IFN-stimulated genes (GBP, IRF1, RSAD2), chemokines (CCL4, CCL4-2, CCL19), matrix metalloproteinase 1 (MMP1), IL-10, and suppressor of cytokine signaling family (SOCS1 and SOCS3) (Sandholt et al., 2021a). Upregulated DEGs related to cytokine-cytokine receptor interactions, such as IL-13RA2, CXCL13, CXCR5, GDF15, IL-7R, IL-12RB2, BMP7, TNFRSF11B, and IL-22, were identified at day 4 post-infection in cecal tissues of chickens mixed-infected with *E. acervulina*, *E. maxima*, and *E. tenella* (Kim et al., 2022). Wenchang chickens (WCC) and recessive white feather chickens (RWFC) have shown different susceptibility to *E. tenella* infection, with WCC being more resistant to the disease and RWFC being more susceptible. In DEG analysis of cecal tissue from *E. tenella*-infected chickens, WCC exhibited a stable immune response, whereas RWFC exhibited a fluctuating pattern. Furthermore, WCC had higher CD3^+^CD4^+^ lymphocyte counts and lower CD3^+^CD8α^+^ lymphocyte counts than RWFC (Li et al., 2025a). Studies of host protective immune responses to *Eimeria* infections have been delayed for a number of reasons, including the complex life cycle of *Eimeria* species in the gut, their intracellular parasitic nature, the presence of multiple *Eimeria* species including precocious strains, chicken breeds with varying susceptibility, the limited availability of *in vivo* studies to date, interactions with gut microbiota, differences in physiological and histological characteristics across regions of the gut, and the deep involvement of intestinal immunity in protective immunity. Although information on the host immune response to *Eimeria* is currently very limited, next-generation sequencing (NGS) and bioinformatics, which have recently become increasingly utilized, are expected to further deepen our understanding of the host immunological aspects of *Eimeria*.

In conclusion, immunological aspects against *Eimeria* infection can be influenced by several factors including chicken breeds (susceptible *vs*. resistant), infection dose of sporulated oocysts, and *Eimeria* species. Orally infected oocysts release sporozoites with the help of digestive enzymes and mechanical movement in the intestine. Free sporozoites pass through the host’s first line of defense, such as physical and chemical barriers including intestinal microflora and mucosa, and then invade epithelial cells, triggering an immune response. The innate immune response is the host’s rapid, non-specific first line of defense, triggered within hours of infection. Innate immune cells utilize PRRs, such as TLRs, C-type lectin receptors (CLRs), cytoplasmic NOD-like receptors (NLRs) and RIG-I-like receptors (RLRs), to detect pathogen-associated molecular patterns (PAMPs) and damage-associated molecular patterns (DAMPs) from damaged cells, initiating pathogen destruction through inflammation, phagocytosis, and the complement system (Wang et al., 2024a). Research on innate immunity to sporozoite infection is very limited, primarily focusing on TLRs, NK-lysin, and profilin. Therefore, more extensive research is needed to improve our understanding of innate immunity using chicken breeds that exhibit different susceptibilities to control coccidiosis infections. Another important factor in preventing chicken coccidiosis is the adaptive immune response. Studies in chickens vaccinated with single antigens, multiple antigens, killed or live oocysts have shown that intestinal mucosal immunity is important and that cell-mediated immunity (CMI) plays a critical role than humoral immunity in *Eimeria*-infected chickens. During these studies, it was considered that IFN-γ may play a positive role in coccidia defense, while IL-17A may play a negative role. The use of live vaccines rather than recombinant vaccines is gradually increasing in the field to reduce the risk caused by *Eimeria* infection in chickens. Therefore, to enhance the effectiveness of live vaccines, it is crucial to identify PAMPs present in *Eimeria* species that can enhance innate immunity.

## 6 Coccidiosis control strategies

### 6.1 Farm management

Effective control of coccidiosis should not depend solely on anticoccidial drugs or vaccination (Peek and Landman, 2011; López-Osorio et al., 2020). A multidisciplinary approach, including environmental management, can suppress oocyst proliferation and transmission, promote uniform flock immunity, and maintain intestinal stability (McDougald, 2020), whereas abrupt environmental fluctuations can lead to uneven exposure and localized outbreaks of various pathogens (Williams, 2005). Moisture and temperature directly determine the sporulation rate and survival of *Eimeria* oocysts. Under optimal conditions of 25–30 °C and 70–90% relative humidity, the unsporulated oocysts of most *Eimeria* species acquire infectivity by forming sporulation within 1–2 days (Mesa-Pineda et al., 2021), while temperatures above 35 °C and freezing temperatures are lethal for oocysts (Peek and Landman, 2011). Excessive litter moisture leads to rapid accumulation of infective oocysts and ultimately increases the risk of clinical coccidiosis by exposing unimmunized young chicks to high doses of sporulated oocysts, whereas dry, well-ventilated litter delays sporulation (Williams, 1998; Mesa-Pineda et al., 2021). Moist areas around drinkers serve as centers of oocyst sporulation. Therefore, litter levelling and adequate ventilation should be applied to minimize the formation of hotspots (Conway and McKenzie, 2007). These practices spatially disperse oocysts, promote low-dose trickle exposure, and help to achieve uniform immunity across the flock (Williams, 2005). There are different opinions regarding the effect of humidity on sporulation and prevalence of coccidiosis. Sporulation of *E. maxima* was more efficient in the dried litter condition (16% moisture content) than in the moisture litter condition (62% moisture content) (Waldenstedt et al., 2001). Flocks in the Pearl River Delta of Guangdong Province, China, where rainfall is high (149 mm) and humidity is relatively low (57%), had a higher prevalence of *E. tenella* than flocks in eastern Guangdong Province, where rainfall is low (118 mm) and humidity is relatively high (approximately 73%). Both areas had similar average annual temperatures of 23–24 °C (Liao et al., 2024). In contrast, in Pakistan, the prevalence of coccidiosis was highly correlated with temperature (25–35 °C) and relative humidity (60–80%) (Awais et al., 2012). Based on 41 articles, the overall prevalence was highest in humid subtropical climate regions, with an average of 75.8%, and ranged from 46.6% to 95.9% (Badri et al., 2024). It is interesting to note that the most important epidemiological aspect during a flock cycle is the time of onset of sporulation, which is influenced by temperature and humidity and is more important than sporulation percentage (Graat et al., 1994).

Bedding and litter conditions are other important factors affecting the outbreak and prevalence of coccidiosis. Recycled litter, including bird droppings, feed scraps, water, and feathers, has a complex microbial composition that varies greatly depending on temporal and spatial differences, which may influence coccidia development. The type and thickness of the litter also influence litter quality, including moisture retention. Rice husks and coarse wood shavings maintain low humidity, while fine sawdust retains more moisture and can facilitate sporulation (Soliman et al., 2018). Oocyst sporulation decreases when litter pH is about 5.3~5.7, which provides a basis for using acidic additives as supplementary control tools (Soliman et al., 2018). There are conflicting results regarding the effects of bedding recycling on fecal oocyst production and chicken productivity depending on litter management. Reused litter with good management showed reduced oocyst numbers in litter samples and improved broiler performance (Garcés-Gudiño et al., 2018), while with natural ventilation, coliform levels, oocyst counts, and litter moisture increased in recycled litter (Stanley et al., 2004). In addition, the maximum sporulation percentage was higher in feces mixed with substrate than in pure feces (Graat et al., 1994). These results indicate that good management of recycled litter is an important factor in controlling coccidiosis outbreaks. In vaccinated flocks, litter serves as the substrate for the secondary and tertiary cycling of vaccine oocysts, inducing protective immunity. Excessive dryness delays recycling, while excessive humidity may lead to runaway recycling and clinical outbreaks (Chapman et al., 2002). Therefore, it is recommended that litter moisture content be maintained, approximately 25–35%, with uniform distribution throughout the chicken house (Grimes and Williams, 2002).

Ventilation affects litter moisture content and gas accumulation, which influence the rate of oocyst sporulation. Low humidity tends to inhibit the recycling of coccidia vaccines and delay immune development, while high humidity can accelerate sporulation and cause early infection (Chapman et al., 2002). Under high temperature and high humidity, this may lead to runaway recycling (Williams, 1998). High stocking densities increase fecal moisture content and accelerate the onset of *Eimeria* infection. Therefore, it is crucial to maintain appropriate stocking density and ventilation to control outbreaks of coccidiosis in poultry farms (Williams, 1999).

Feed and water intake patterns significantly influence the degree of oocyst recycling and the uniformity of flock immunity (Shirley et al., 2005). When chickens repeatedly intake feed or water in restricted areas, high oocyst concentrations are formed, resulting in overexposure in some individuals and ultimately lowering the uniformity of immune acquisition throughout the flock (Williams, 2005). For flocks vaccinated by spraying or watering, uniform vaccine uptake in the hatchery or on-farm is also crucial. Because vaccines rely on repeated recycling infections to induce protective immunity, inconsistent vaccine uptake may lead to delayed infection or incomplete immunity in certain individuals (Williams, 1998; Shirley et al., 2005).

Sufficient downtime with drying and cleaning between flocks is essential to reduce environmental oocyst load (Chapman, 1997; Conway and McKenzie, 2007). Even with vaccination, a downtime shorter than 10 days can lead to coccidiosis outbreaks because field strains may infect chicks before vaccine oocysts can recycle, resulting in vaccine bypass infection (Conway and McKenzie, 2007). The major farm management-related determinants influencing environmental oocyst pressure are summarized in **Table 2**. On the other hand, most disinfectants are ineffective against oocysts; therefore, mechanical cleaning and drying remain the most effective measures (Mathis and McDougald, 1982). Previous studies have tested the effects of various chemicals on their oocysticidal effect, but efficacy is highly agent-specific and concentration-dependent with significant variation across different chemicals and disinfectant classes (**Table 3**). While certain laboratory-grade agents or targeted organic acids show high sporulation inhibition, many common commercial multicomponent disinfectants provide negligible protection against *Eimeria*. In general, since the temperature of the poultry house is controlled within a certain range while the chickens are growing, it is important to maintain the moisture content of the bedding at a low level to reduce the sporulation and survival rate of *Eimeria* oocysts.

**Table 2:** Farm management-related factors influencing *Eimeria* transmission and coccidiosis risk in poultry production.

Category	Parameter/factor	Impact on pathogen and host dynamics	References
Microclimate	Temperature	▪ 25–30 °C: optimal sporulation ▪ >35 °C and freezing: lethal for oocyst	Peek and Landman, 2011; Mesa-Pineda et al., 2021
	Relative humidity	▪ 70–90%: optimal sporulation ▪ Low humidity: inhibit recycling of coccidia vaccine	Mesa-Pineda et al., 2021
Litter	Substrate type	▪ Rice husks and coarse wood shavings: low retention of moisture ▪ Fine sawdust: high retention of moisture	Soliman et al., 2018
	pH	▪ pH 5.3~5.7: decrease sporulation	Soliman et al., 2018
Management	Stocking density	▪ High stocking: increase fecal moisture and accelerate onset of *Eimeria* infection	Williams, 1999
	Downtime	▪ <10 days: risk of coccidiosis vaccine bypass by field strain	Conway and McKenzie, 2007
Biosecurity	Sanitation	▪ Most are ineffective against *Eimeria* spp. oocyst	Mathis and McDougald, 1982

**Table 3:** Comparative efficacy of chemical agents and disinfectant classes on the sporulation and structural integrity of *Eimeria* spp. oocysts.

Class	Example chemical agent	Concentration and impact on *Eimeria* spp. oocyst	Comments on its applications and/or limitations of use	References
Alcohol	Ethanol (EtOH)	▪ 20%: 2.7% sporulation inhibition and mild oocyst wall deformities after 48h ▪ 50%: 52.4% sporulation inhibition, marked oocyst wall deformities after 48h ▪ 70%: total inhibition of mix *Eimeria* spp. oocyst sporulation and deterioration of oocysts wall after 48h	Flammable, skin toxicity in high alcohol concentration and short half-life as microbial agent	Gadelhaq et al., 2018; Alajlan et al., 2022
Aldehydes	Formalin	▪ 5%: 3.4% sporulation inhibition, mild oocyst wall deformities after 48h ▪ 10%: total inhibition of mix *Eimeria* spp. oocyst sporulation after 48h	Toxicity and health hazards	Gadelhaq et al., 2018; Santos et al., 2025
Aromatic hydrocarbons	Benzene (39%) + Xylene (22%)	▪ 1:10 dilution: 75.9% sporulation inhibition of *E. tenella* oocysts at 30 mins	Highly toxic and hazardous to health, not for practical application	You, 2014; Saeedi et al., 2024
Base	Sodium hydroxide (10N)	▪ 1:1 dilution: ineffective in inhibiting sporulation of *E. tenella* oocysts at 30 mins	Airway irritant	You, 2014; Addie et al., 2015
	Potassium hydroxide (KOH)	▪ 50mg/mL: 30.5% sporulation inhibition of *E. tenella* oocysts after 48h	Not effective against *Eimeria* oocyst	Abd-Elrahman et al., 2022
Carbamate	Carbamate type 88%	▪ 1:8 dilution: <50% sporulation inhibition of *E. tenella* oocysts at 30 mins ▪ 1:88 dilution: <20% sporulation inhibition of *E. tenella* oocysts at 30 mins	Used primarily as insecticides, can accumulate to environment, lack selectivity to targets, toxic and can cause poisoning	Meerdink, 1989; You, 2014; Singh et al., 2025
Halogen-based compound	Sodium hypochlorite (NaOCl)	▪ 2ml 0.5% in 5ml solution: 48.32% of mix *Eimeria* spp. oocyst sporulation after 48h, oocyst wall breakage and corrugation ▪ 1:10 dilution: <10% sporulation inhibition of *E. tenella* oocysts at 30 mins	Common household bleach, airway irritants	You, 2014; Addie et al., 2015; Gadelhaq et al., 2018
Multicomponent disinfectants	TH4+, Virkon^®^S, Dettol	▪ 25µl TH4+ or 5mg Virkon^®^S or 109µL Dettol in 5ml solution: did not inhibit sporulation of mix *Eimeria* spp. oocyst sporulation after 48h	Not effective against *Eimeria* oocyst	Gadelhaq et al., 2018
Organic acid	Acetic acid 99.9%	▪ 1:2 dilution: 91.7% sporulation inhibition of *E. tenella* oocysts at 30 mins	Readily available but pungent, irritating odor	You, 2014; Addie et al., 2015
	Malic acid 10%	▪ 1:10 dilution: <20% sporulation inhibition of *E. tenella* oocysts at 30 mins	Not effective against *Eimeria* oocyst	You, 2014
Oxidizing agent	Aqueous ozone	▪ 4.93mg/L: 93% sporulation inhibition of *E. acervulina* sporulation by day 7	Potent antibacterial agent, safe to use in meat and poultry production	Megahed et al., 2020; Baumann et al., 2024
	Hydrogen peroxide	▪ 3%: <40% sporulation inhibition of *E. tenella* oocysts at 30 mins	Not stable and dissociates	You, 2014; Addie et al., 2015
Phenolics	Cresol soap	▪ 30%: 85.5% sporulation inhibition of *E. tenella* oocysts at 30 mins	Effectivity vary substantially depending on formulation	McLaren et al., 2011; You, 2014
Quaternary ammonium	Quaternary ammonium salt 5%	▪ 1:5 dilution: <40% sporulation inhibition of *E. tenella* oocysts at 30 mins ▪ 1:50 dilution: <10% sporulation inhibition of *E. tenella* oocysts at 30 mins	Inactivated by organic materials, soap and water; extensive use may lead to emergence of antibiotic-resistant bacteria, Toxicity and health hazards, aquatic environment hazard	You, 2014; Addie et al., 2015; Kim et al., 2020
Quaternary ammonium with aldehyde	Quaternary ammonium salt 6%+Aldehyde 13%	▪ 1:10 dilution: <20% sporulation inhibition of *E. tenella* oocysts at 30 mins	Toxicity and health hazards, aquatic environment hazard	You, 2014; Kim et al., 2020

### 6.2 Anticoccidial drugs

Anticoccidial drugs have been the primary means to prevent and manage coccidiosis disease in poultry for more than a century (Chapman, 2001; Nawarathne et al., 2021). The journey of anticoccidial drug development and application reflects a continuous effort to prevent the evolving *Eimeria* challenge. Research into anticoccidial drugs gained significant momentum in the late 1940s, and the first related publication on the anticoccidial activity of nitrophenide, by Waletzky, Hughes, and Brandt, appeared in 1949 (Chapman, 2001; Hafez, 2008; Noack et al., 2019; Nawarathne et al., 2021; Kandeel et al., 2023; Blake, 2025). This represented a crucial transition from the management of overt diseases to the implementation of ongoing preventive medication (Chapman and Rathinam, 2022). The ideal anticoccidial drug is characterized by high efficacy, broad spectrum activity against various *Eimeria* species, a large therapeutic index with a wider range of safe dosages, cost effectiveness, and the ability to be metabolized as well as excreted without toxic residuals (Kandeel et al., 2023).

In general, anticoccidial drugs can be primarily classified into two main categories: polyether ionophores and synthetic chemical compounds (Kandeel et al., 2023; Ahmad et al., 2024; Mathis et al., 2025). Ionophores are natural compounds produced by the fermentation of bacteria such as *Streptomyces* spp. or *Actinomadura* spp. (Noack et al., 2019; Nawarathne et al., 2021; Martins et al., 2022; Ahmad et al., 2024) and act by disrupting ion gradients across the parasite cell membrane through selective permeability to monovalent cations of sodium ions (Na^+^), potassium ions (K^+^), rubidium ions (Rb^+^), cesium ions (Cs^+^), and divalent cations of calcium ions (Ca^2+^), barium ions (Ba^2+^), and magnesium ions (Mg^2+^), ultimately leading to cellular dysfunction and parasite death (Quiroz-Castañeda and Dantán-González, 2015; Noack et al., 2019; Ahmad et al., 2024). Ionophores were subclassified into monovalent ionophores (monensin, narasin and salinomycin) (Chapman, 2001; Kadykalo et al., 2018; Noack et al., 2019; Ahmad et al., 2024; Sun et al., 2025; Tongkamsai et al., 2025), monovalent glycosidic ionophores (maduramicin and semduramicin) (Hafez, 2008; Noack et al., 2019), and divalent ionophores (lasalocid) (Noack et al., 2019; Ahmad et al., 2024; Tongkamsai et al., 2025). Ionophores have narrow safety margins, with toxicity expressed as median lethal dose (LD_50_) only 10 – 20% above therapeutic levels (Ekinci et al., 2023).

Different classes of ionophores have significantly selective profiles for metal cations, leading to distinct patterns of disruption in the sporozoite and merozoite stages of the parasite (Smith and Strout, 1980; Mehlhorn et al., 1983; Conway et al., 1993; Wang et al., 2006; Noack et al., 2019; Ahmad et al., 2024; Zhao et al., 2024). Monensin demonstrated a strong preference for monovalent cations, with a marked inclination for binding to Na^+^ over K^+^. This selectivity results in an increase in the intracellular concentrations of both Na^+^ and K^+^. The proposed mechanism involves the initial uptake of Na^+^, followed by an exchange process in which intracellular Na^+^ is exchanged for extracellular K^+^ (Frederiksen et al., 2024). Monensin, which is a fermentation product of *Streptomyces cinnamonensis* approved as a coccidiostat (Chapman et al., 2010), is the most extensively used ionophore globally. In the poultry industry, monesin demonstrated effective and consistent activity against most *Eimeria* species (Chapman et al., 2010; Antoszczak et al., 2019). The concentration used for coccidiosis prevention often includes 100 ppm or 60–125 ppm added to feed (Long and Millard, 1978; Bello et al., 2023). When used in synergistic combination with nicarbazin, the effective dose included 40 ppm monensin and 40 ppm nicarbazin (Chapman and Rathinam, 2022). The mechanism of action described by Martins et al., 2022 is that monensin acts as a carrier and transports ions Na^+^ and K^+^ across the hydrophobic membranes of the *Eimeria* parasite, resulting in increased intracellular ion concentrations and osmotic imbalance and causing the parasite to vacuolize, swell, and burst (Martins et al., 2022). A pharmacokinetic study reported extremely low systemic bioavailability of monensin, with approximately 3.9% absolute bioavailability, reflecting significantly limited gastrointestinal absorption (Henri et al., 2017). Despite minimal systemic absorption, monensin remains effective because its antimicrobial action occurs directly within the intestinal lumen and epithelial cells, where feed-mediated application sustains a high local drug concentration (Chapman et al., 2010).

Narasin, a polyether ionophore generated by fermentation of *Streptomyces aureofaciens*, is commonly employed as an anticoccidial feed additive in poultry to prevent and manage coccidiosis caused by *Eimeria* species with a range of treatment dose from 60 to 70 mg/kg (Ruff et al., 1979, 1980; Jeffers et al., 1988; Additives, E.Panel o et al., 2018a, 2024). Narasin, a salinomycin derivative with an additional methyl group ((4S)-4-methyl salinomycin), exhibits intrinsic binding selectivity for K+ over Na^+^, thereby disrupting transmembrane ion gradients and triggering osmotic-induced cell death (Frederiksen et al., 2024). Beyond its anticoccidial activity, narasin promotes gut health by reducing the prevalence of *Clostridium perfringens* and *necrotic enteritis* (Laurentie et al., 2023; Rogala-Hnatowska et al., 2024). Comparative studies have demonstrated that it is at least as effective as, and sometimes superior to, other ionophores such as salinomycin and monensin, particularly in enhancing growth performance, intestinal integrity, and production efficiency in broiler chickens subjected to *Eimeria* species challenge (Ruff et al., 1980; Jeffers et al., 1988; Lei et al., 2022; Rogala-Hnatowska et al., 2024).

Salinomycin exhibits a notable selectivity for alkali ions, demonstrating a strong preference for K^+^ over Na^+^. This preferential binding facilitates the efflux of K^+^ from mitochondria, which subsequently leads to mitochondrial dysfunction. The resulting effects include alterations in membrane potential, inhibition of oxidative phosphorylation, and disruption of cellular energy balance, culminating in cell death (Kim et al., 2012; Choi et al., 2013). The variations in selectivity result in distinct patterns of cellular disruption, despite the overarching similarity in the mechanism class. Salinomycin, which is produced from *Streptomyces albus* is commonly incorporated into feed at concentrations of 60 to 70 ppm, has been demonstrated to be a highly effective anticoccidial agent in poultry. It includes benefits such as reduced oocyst shedding, decreased mortality rate, suppressed lesion scores, improved body weight gain, and improved feed conversion ratio (Danforth et al., 1977a, b; McDougald et al., 1981; Spišáková et al., 2024). With performance comparable to other ionophores such as monensin and narasin, some data illustrated that salinomycin shows higher improvement of chicken growth performance in certain settings (Danforth et al., 1977b; Rogala-Hnatowska et al., 2024). Salinomycin disrupts ion gradients across the parasite membrane, impairing *Eimeria* cellular function and survival and targeting the asexual propagation stages of *Eimeria* species (Chappel, 1979; Qaid et al., 2021). Nevertheless, the widespread utilization of salinomycin has precipitated the development of resistant *Eimeria* strains. This has consequently diminished the drug’s effectiveness and underscored the necessity for comprehensive control measures, including drug rotation and the integration of vaccination strategies to reestablish drug sensitivity (Chapman and Jeffers, 2015; Flores et al., 2022). A recent study has identified specific genetic mutations, such as the F204S substitution in adrenodoxin oxidoreductase, that drive salinomycin resistance in *E. tenella* (Sun et al., 2024), leading to a need for ongoing surveillance and novel control approaches.

Semduramicin, a polyether ionophore produced by fermentation of *Actinomadura* spp., is an effective anticoccidial drug widely used in poultry for controlling coccidiosis (Additives, E.Panel o et al., 2018b). The recommended feed dosage for broilers is 20–25 mg/kg, and 25 mg/kg is identified as the optimal dose for balancing lesion score and weight gain in chickens (Ricketts et al., 1992; Logan et al., 1993; McKenzie et al., 1993; Additives, E.Panel o et al., 2018b, 2022a). Consistent with other monovalent ionophores, semduramicin combines more readily with K^+^ and Na^+^ and disrupts ion gradients across the parasite cell membrane, leading to osmotic imbalance and cell death, and demonstrates broad-spectrum efficacy against multiple *Eimeria* species including *E. acervulina*, *E. brunetti*, *E. maxima*, and *E. tenella* (Ricketts et al., 1992; Conway et al., 1993; Logan et al., 1993; Conway et al., 1995; Anadón and Martínez-Larrañaga, 2014; Frederiksen et al., 2024). Semduramicin is generally well tolerated in broilers when administered at recommended doses, and exhibits no significant adverse effects on growth performance, feed efficiency, or primary hematological and biochemical parameters, though some studies have reported mild immunosuppressive effects, including decreased phagocytic activity and serum globulin level (Dakroury and Resik, 2013; Additives, E.Panel o et al., 2018b). Additionally, semduramycin can be combined at reduced doses with other ionophores, such as salinomycin, to achieve effective coccidiosis control and improved performance outcomes (Kraieski et al., 2021; Chapman and Rathinam, 2022).

Maduramicin, a monoglucoside polyether produced through the fermentation of *Actinomadura* spp., is a highly potent anticoccidial agent incorporated into feed at a recommended dosage of 5 mg/kg for broiler chickens, and has been demonstrated to significantly enhance growth performance, feed conversion ratio, and survival rate, while also decreasing oocyst shedding and lesion scores in birds infected with *Eimeria* species (McDougald et al., 1987; Shashi Kala et al., 2014; Attia et al., 2021). Maduramicin inhibits *Eimeria* infection by disrupting transmembrane ion gradients in *Eimeria* parasites, leading to osmotic imbalance and cell death. This is achieved by its ability to form complexes with cations such as Na^+^ and K^+^, facilitating their transport across the parasite’s cell membrane and collapsing essential electrochemical gradients (Chen et al., 2014; Abdelhady et al., 2021). Recent mechanistic studies demonstrated that maduramicin can induce both caspase-dependent and -independent apoptosis in host myocardial cells, involving increased intracellular calcium, mitochondrial dysfunction, and activation of apoptotic pathways, as well as non-apoptotic cell death by methuosis-like cytoplasmic vacuolization (Chen et al., 2018; Gao et al., 2018, 2020). These findings provide biological rationale for the cardiotoxicity associated with maduramicin and underscore the necessity of strict industrial safety protocols to mitigate risks to non-target species.

Lasalocid is commonly classified as a divalent polyether ionophore because it forms stable complexes with divalent cations, particularly Ca²^+^ and Mg²^+^, although it can also complex monovalent cations such as Na^+^ and K^+^. Cellular disruption has been demonstrated to be associated with alterations in ionic homeostasis, including aberrations in Ca²^+^ handling (Mitani and Otake, 1978; Antonio et al., 1991; Anadón and Martínez-Larrañaga, 2014; Frederiksen et al., 2024). Lasalocid is a well-established anticoccidial agent typically used at 75 to 90 mg/kg of feed in the poultry industry, and demonstrates strong efficacy against various *Eimeria* species by reducing lesion scores, oocyst shedding, and mortality rate, while supporting normal body weight gain and feed conversion efficiency (Mitrovic and Schildknecht, 1974; 1975; Additives, E.Panel o et al., 2022b).

In floor pen and battery trials using a mixture of five *Eimeria* species, lasalocid administered at doses ranging from 75 to 125 ppm effectively alleviated severe morbidity and mortality, and completely prevented lesion scores, particularly at the higher dose of 125 ppm, although the optimal dose was observed to be between 75 and 100 ppm (Bains, 1980). Recent regulatory reviews have established 90 mg/kg as an effective and safe level for chickens for fattening, although a margin of safety could not be confirmed for chickens reared for laying, and caution is advised due to potential residue accumulation in eggs, especially considering breed-dependent differences in lasalocid pharmacokinetics (Additives, E.Panel o et al., 2022b; Bello et al., 2024). Mechanistically, lasalocid disrupts cation transport across the *Eimeria* membrane and impairs ion gradients and energy metabolism, which leads to parasite death (Ahmad et al., 2024). Histomorphological studies further corroborated the protective effects of lasalocid by demonstrating the preservation of intestinal villi structure and growth performance in *E. tenella*-infected chickens (Giannenas et al., 2014; Zarei and Shahhosseini, 2023).

Generally, the efficacy and safety of polyether ionophores are maintained through standardized industrial frameworks. Parasite sensitivity to drugs is preserved by utilizing shuttle or rotation programs, often alternating ionophores with drug-sensitive live vaccine strains to restore the efficacy of the anticoccidials (Noack et al., 2019; Vereecken et al., 2021; Chapman and Rathinam, 2022; Glorieux et al., 2022). Field safety relies on the avoidance of concurrent administration with contraindicated drugs, such as tiamulin, to prevent lethal metabolic interference (Laczay et al., 1990; Badiola et al., 1994; Rychen et al., 2018; Noack et al., 2019; Bampidis et al., 2024; Gómez et al., 2025). Additionally, mixer quality assurance and feed-line sequencing are employed to prevent drug carryover or cross-contamination that poses health risks to non-target species, while strict compliance with withdrawal periods ensures regulatory and food safety (Anadón and Martínez-Larrañaga, 2014). A summary of each polyether ionophore discussed above, and their pharmacological profiles and strategic applications are presented in **Table 4**.

**Table 4:** Summary profile and strategic use of polyether ionophores for coccidiosis control.

Ionophore generic name /source organism	Ionophore classification	Target parasite stage	Selectivity of ions	Advantages	Limitations/field mitigation measure	Concentration in feed	Stage of use in poultry	References
Monensin (*Streptomyces cinnamonensis)*	Monovalent ionophore	Sporozoite, merozoite	Na^+^, K^+^	Improve growth performance, slow development of resistance, prevents clinical effects of coccidiosis while allowing birds to acquire immunity, synergistic combination with nicarbazin	Low systemic bioavailability, documented resistance in *Eimeria* spp. isolates, toxicity risk at high dose/avoid concurrent use with tiamulin, use as part of rotation or shuttle program, alternate with drug sensitive live vaccine strain	100 ppm, 60–125 ppm	Starter feed: occasionally or as part of shuttle program with another synthetic drug; Grower feed: most common; First withdrawal feed: occasionally; lay chicken: only in pullets	Long and Millard, 1978; Smith and Strout, 1980; Mehlhorn et al., 1983; Wang et al., 2006; Chapman et al., 2010; Henri et al., 2017; Noack et al., 2019; Qi et al., 2020; Vereecken et al., 2021; Chapman and Rathinam, 2022; Bello et al., 2023; Frederiksen et al., 2024
Narasin (*Streptomyces aureofaciens)*	Monovalent ionophore	Sporozoite	K^+^, Na^+^	Improve growth performance, effective in reducing *Clostridium perfringens* and necrotic enteritis,	Documented resistance in *Eimeria* spp. isolates, potential terrestrial ecotoxicity/avoid concurrent use with tiamulin, use as part of rotation or shuttle program, alternate with drug sensitive live vaccine strain	60–70 mg/kg	Starter feed: as part of shuttle program with another synthetic drug; Grower-finisher feed: most common	Smith and Strout, 1980; Long et al., 1988; Badiola et al., 1994; Anadón and Martínez-Larrañaga, 2014; Noack et al., 2019; Vereecken et al., 2021; Bampidis et al., 2024; Frederiksen et al., 2024; Rogala-Hnatowska et al., 2024
Salinomycin (*Streptomyces albus)*	Monovalent ionophore	Merozoite, sporozoite	K^+^, Na^+^	Improve growth performance, has inhibitory effect to *Clostridium perfringens*, low residue level in the eggs compared to other polyether ionophores, least toxic of all ionophores, combination with nicarbazin	Documented resistance in *Eimeria* spp. isolates/avoid concurrent use with tiamulin, alternate with drug sensitive live vaccine strain, use as part of rotation or shuttle program	60–70 ppm	Broiler: grow-out period; Lay chickens: only in pullets	Conway et al., 1993; Mehlhorn et al., 1983; Badiola et al., 1994; Anadón and Martínez-Larrañaga, 2014; Zavala et al., 2018; Noack et al., 2019; Vereecken et al., 2021; Flores et al., 2022; Frederiksen et al., 2024
Semduramicin (*Actinomadura* spp.*)*	Monovalent glycosidic ionophore	Sporozoite	K^+^, Na^+^	Highly effective and well tolerated in chickens, broad spectrum efficacy against multiple *Eimeria* spp., synergistic effect with salinomycin at reduced doses	Mild immunosuppresive effects, not effective against gram negative bacteria, risk of aquatic ecotoxicity and groundwater pollution/avoid concurrent use with tiamulin, alternate with drug sensitive live vaccine strain, use as part of rotation or shuttle program	20–25 mg/kg	Broiler: grow-out period	Conway et al., 1993; Anadón and Martínez-Larrañaga, 2014; Rychen et al., 2018; Noack et al., 2019
Maduramicin (*Actinomadura* spp.*)*	Monovalent glycosidic ionophore	Sporozoite, merozoite	K^+^, Na^+^	Highly potent anticoccidial agent, can inhibit growth of gram-positive microorganisms	Documented resistance in *Eimeria* spp. isolates, most toxic documented ionophores for humans and non-target animals/avoid concurrent use with tiamulin, withdrawal period compliance, use as part of rotation or shuttle program, strict compliance to industrial safety protocol to avoid toxicity in non-target species	5 mg/kg	Broiler: grow-out period	Laczay et al., 1990; Anadón and Martínez-Larrañaga, 2014; Noack et al., 2019; Frederiksen et al., 2024; Zhao et al., 2024
Lasalocid (*Streptomyces lasalocidi* sp. *nov.)*	Divalent ionophore	Merozoite, sporozoites	K^+^, Na^+^, Ca^2+^, Mg^2+^	High selectivity to intracellular sporozoites, safe to host cells, high efficacy to various *Eimeria* spp.	Documented resistance in *Eimeria* spp. isolates, require longer withdrawal period and produce highest residue concentration in eggs any other polyether compounds, lasalocid carryover or cross contamination found in various feed batches pose health risk to nontarget species/withdrawal period compliance, feed line sequencing and mixer QA, use as part of rotation or shuttle program	75–90 mg/kg	Broiler: grow-out period; Lay chickens: only in pullets	Mehlhorn et al., 1983; Anadón and Martínez-Larrañaga, 2014; Noack et al., 2019; Frederiksen et al., 2024

Synthetic compounds, including nicarbazin, toltrazuril, diclazuril, and amprolium, which are primarily used on farms, were produced through chemical synthesis. Nicarbazin, which was introduced in 1955, was recognized as the first broad-spectrum anticoccidial drug, thus holding considerable historical significance in the field of veterinary medicine with extensive use in broiler production (Hafez, 2008; Noack et al., 2019). The 1970s saw the launch of several other highly efficacious synthetic drugs, followed by the groundbreaking arrival of ionophores (Noack et al., 2019; Nawarathne et al., 2021). Nicarbazin has achieved notable success as an anticoccidial agent that is commonly employed to block schizont development during both the first and second generations in the *Eimeria* life cycle (Quiroz-Castañeda and Dantán-González, 2015). However, it cannot be used to control *Eimeria* in laying hens due to its negative effect on egg production at approved treatment concentrations in poultry (Chapman and Rathinam, 2022). Nicarbazin is commonly administered to broiler chickens at dosages of 100 and 125 mg/kg in feed, often as part of combination products with other ionophores such as narasin or monensin to increase efficacy and mitigate the development of resistance (Abdelhady et al., 2021; Chapman and Rathinam, 2022). A previous study reported that combination of nicarbazin and salinomycin at 40ppm improved the body weight gain, feed conversion and productive efficiency index of *Eimeria* infected broilers (Zavala et al., 2018). The mechanism of action of nicarbazin remains incompletely understood; however, it is believed to interfere with the development of *Eimeria* species by disrupting energy metabolism and inhibiting amylopectin synthesis, which are crucial for parasite replication and oocyst wall formation (Abdelhady et al., 2021). There is a broad range of efficacy against major *Eimeria* species including *E. acervulina*, *E. maxima*, and *E. tenella*, and it is effective in both laboratory and field conditions, particularly when used in rotation or a combination program (Abdelhady et al., 2021; Tongkamsai et al., 2025). On the other hand, a recent report using *Eimeria* spp. field isolates from 10 European countries showed poor sensitivity of the isolates from farms using in-feed anticoccidial programs to a combination of narasin and nicarbazin (Glorieux et al., 2022). Resistance can develop with prolonged use, leading to strategic rotation and recombination with other anticoccidial drugs (Abdelhady et al., 2021; Tongkamsai et al., 2025).

Toltrazuril is given to broiler chickens with drinking water at a dose of 25 ppm for a duration of two days, and the dosage varies from 25 to 75 mg/kg depending on the severity of *Eimeria* infection (Ramadan et al., 1997; Ojimelukwe et al., 2018). Toltrazuril is recognized for its high efficacy against a wide range of *Eimeria* species, including *E. tenella*, *E. acervulina*, and *E. maxima*. Its use results in a significant reduction in oocyst output and an observable decrease in lesion scores (Ramadan et al., 1997; Ojimelukwe et al., 2018). The mechanism of action of toltrazuril involves the disruption of nuclear division and mitochondrial function within *Eimeria*, leading to the inhibition of respiratory chain enzymes and hindering parasite development at various intracellular stages (Ramadan et al., 1997). Recent reports have highlighted the emergence of resistance to toltrazuril in field isolates. This resistance has been evaluated with the anticoccidial index and various outcome parameters, including weight gain, feed intake, lesion scores, and oocyst shedding (Lan et al., 2017; Ojimelukwe et al., 2018; Flores et al., 2022).

Diclazuril is a benzeneacetonitrile compound based on reizine that is commonly used at 1-2.5 ppm in feed or drinking water for the control of major pathogenic *Eimeria* species in broiler and layer chickens in poultry farms (Vanparijs et al., 1989; Mcdougald et al., 1991; Amer et al., 2007; El-Sawah et al., 2024). Diclazuril inhibits the development of *Eimeria* by specifically targeting the depolymerizing factor involved in actin filament turnover. This disruption impairs cytoskeletal dynamics that are essential for parasite motility, host cell invasion, and intracellular replication. Consequently, this results in the inhibition of both sexual and asexual stages, a reduction in oocyst replication, and the destruction of intracellular schizonts and gamonts (Abd El Monsef et al., 2024; Tian et al., 2025). However, increasing reports of resistance in field isolates and reduced sensitivity in some regions underscore the need for integrated control programs and rotation strategies (Mesa et al., 2021; Flores et al., 2022).

Amprolium functions as an inhibitor of thiamine uptake in second-generation schizonts of *E. tenella* (Quiroz-Castañeda and Dantán-González, 2015). This agent is typically used at 125 mg/kg in feed or 100 ppm in drinking water for 5–7 days. As a thiamine analogue, it competitively inhibits thiamine uptake in *Eimeria*, thereby disrupting carbohydrate metabolism and inhibiting schizogony, ultimately starving the parasite and preventing growth and reproduction (Ojimelukwe et al., 2018; Abdelhady et al., 2021). Amprolium is particularly effective against *E. acervulina*, *E. necatrix*, and *E. tenella*, with moderate efficacy in both laboratory and field conditions (Ojimelukwe et al., 2018; Chalalai et al., 2025). Resistance has been reported, especially with long-term use, and combination use with sulfonamides or probiotics is being studied to increase efficacy and reduce the development of drug-resistant strains (Ojimelukwe et al., 2018; Chalalai et al., 2025; Mustafa et al., 2025). The summary of the synthetic drug used in poultry was presented in **Table 5**. Collectively, anticoccidial drugs to control coccidiosis have been widely used as feed and drinking water additives in poultry farms, leading to the emergence of antibiotic-resistant *Eimeria* strains and threatening the continued growth of the poultry industry. Furthermore, concerns have been raised about the potential human health risks associated with drug residues in poultry products such as meat and eggs. To reduce these two negative effects, recombinant use and strategic rotation of drugs have been recommended in commercial settings.

**Table 5:** Summary of synthetic anticoccidial drugs used in poultry.

Drug name	Molecular formula	Chemical structure^*^	Concentration in feed (ppm)	Against Eimeria species	Mechanism action	Typical stages target	Advantages	Limitations/field mitigation measures	Used in poultry species	Reference
Amprolium	C_14_H_19_ClN_4_	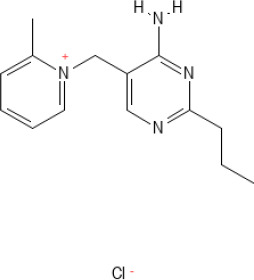	125-250	*E. acervulina, E. necatrix, E. tenella*	Blocks absorption of thiamine in *Eimeria*, blocks carbohydrate metabolism, and cell division	Propylactic, early asexual stage	Wide safety margin, low cost, minimal withdrawal	Narrow spectrum, resistance potential/rotation or shuttle programs, oocyst monitoring	Broiler chickens and turkeys	(Kandeel, 2011; Ojimelukwe et al., 2018; Rychen et al., 2018)
Diclazuril	C_17_H_9_Cl_3_N_4_O_2_	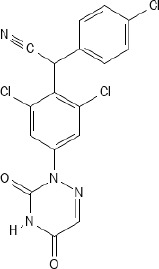	1-2	*E. acervulina, E. maxima, E. tenella*	Inhibits nuclear division of mitosis, and mitochondrial function	Preventive, asexual stages	High potency, broad spectrum	Resistance emergence/Rotation programs, sensitivity testing	Broiler chickens	(Ahmad et al., 2024; Tian et al., 2025; Tongkamsai et al., 2025)
Nicarbazin	C_19_H_18_N_6_O_6_	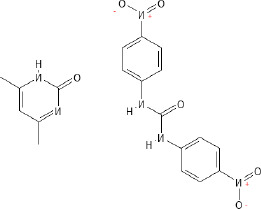	100-125	*E. acervulina, E. brunetti, E. maxima, E. necatrix, E. tenella*	Affecting the second generation of schizonts and gametes, interrupts DNA replication	Mid asexual stages	Broad spectrum efficacy, well characterized residues	Heat stress sensitivity, laying restrictions/ionophore combinations	Broiler chickens and turkeys	(Bafundo et al., 2008; Noack et al., 2019; Rychen et al., 2018; Yoder et al., 2006)
Toltrazuril	C_18_H_14_F_3_N_3_O_4_S	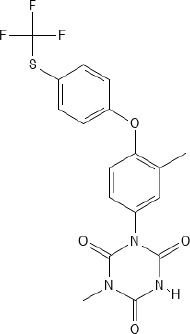	1-25	*E. acervulina, E. maxima, E. tenella*	Disrupt parasite mitochondrial function, induces oxidative stress and cell autophagy, impairs nuclear division and development processes	All stages	High efficacy	High cost, resistance risk/target outbreak treatment, label withdrawal compliance	Broiler chickens	(Kandeel, 2011; Karmakar et al., 2025; Mathis et al., 2003; Ramadan et al., 1997; Zhang et al., 2023)

^*^Chemical structure created through PubChem Sketcher version 2.4.

### 6.3 Vaccines

Negative concerns about the use of prophylactic anticoccidial agents have recently shifted more focus on vaccination. Therefore, information related to recombinant/subunit vaccines or non-attenuated/attenuated vaccines has become increasingly important (Peek and Landman, 2011; Ahmad et al., 2016; Blake et al., 2017; Liu et al., 2023). Approximately 6,000 to 9,000 proteins are expressed throughout the life cycle of *Eimeria* species (Blake et al., 2017; Olajide et al., 2022), many of which, particularly antigens expressed during the sporozoite and merozoite stages, are involved in protective immune responses (Britez et al., 2023; Jebessa et al., 2025). Because genetic or antigenic diversity exists among *Eimeria* species to achieve immune evasion, to avoid anticoccidial drugs, or through natural selection (Kumar et al., 2015; Võ et al., 2021; Adeyemi et al., 2023), recombinant antigens and *Eimeria* strains for vaccines may require periodic monitoring to produce effective vaccines. The comparative summary of the diverse vaccine platforms in coccidiosis in this current paper is presented in **Table 6**, whereas detailed evaluations of conventional and next-generation anticoccidial vaccines, including antigen delivery, delivery platforms, and field applicability are comprehensively reviewed elsewhere (Ahmad et al., 2016; Blake et al., 2017; Nasri et al., 2022; Zaheer et al., 2022; Xu and Li, 2024).

**Table 6:** Summary of anticoccidial vaccines platforms and strategies.

Vaccine type	Description	Evidence level	Key advantages	Limitations	References
Attenuated (precocious) vaccines	Shortened life-cycle *Eimeria* spp. strain	Commercial/field use	Safer than wild-type virulent strains	High production cost, complex manufacturing process	Peek and Landman, 2011; Ahmad et al., 2016; Liu et al., 2023
DNA vaccines/Recombinant proteins	Defined *Eimeria* antigens (i.e. RON2, ROP21, ROP27, EF2, AMA, 14-3-3, glyceraldehyde-3-phosphate dehydrogenase, transhydrogenase)	Experimental	Safe, antigen-specific, multivalent design possible	Injectable, difficult mass application, partial protection	Pastor-Fernández et al., 2018; Mi et al., 2023; Shi et al., 2023; Chen et al., 2023, 2024; Zhao et al., 2025
Inactivated sporozoite vaccine	Inactivated sporozoite in adjuvant	Experimental	High protective efficacy based on ACI	Parenteral delivery, scale-up constraints	Zhao et al., 2019
Live non-attenuated vaccines	Wild type *Eimeria* spp. strain	Commercial/field use	Proven efficacy, restores drug sensitivity, antibiotic-free programs	Require oocyst cycling, performance variability	Peek and Landman, 2011; Ahmad et al., 2016
Vectored vaccines	*Eimeria* antigen expressing bacteria or yeast	Experimental	Mucosal immunity, oral delivery	Stability, dosing, regulatory challenges	Liu et al., 2021; Ma et al., 2021; Pan et al., 2023
Transgenic/gene edited *Eimeria*	Genetically modified *Eimeria* spp. parasites	Proof-of-concept	Multivalent antigen delivery, cross-protection possible	Early development, scalability and regulatory barriers	Pastor-Fernández et al., 2018; Fatoba and Adeleke, 2020; Tang et al., 2020; Cheng et al., 2021; Liu et al., 2023

ACI, anticoccidial index; AMA, apical membrane antigen; EF, elongation factor; RON, rhoptry neck protein; ROP, rhoptry organelle protein.

#### 6.3.1 Recombinant vaccines

Vaccines utilizing antigens include purified recombinant proteins, DNA fragments encoding antigens, or microorganisms expressing antigens. The antigens mainly considered as anticoccidial vaccine candidates are surface or secreted proteins expressed during asexual and sexual stages. Antigens such as microneme (MIC) proteins, rhoptry organelle proteins (ROPs), apical membrane antigen (AMA), glycosylphosphatidylinositol (GPI)-anchored surface antigens (SAGs), immune-mapped protein (IMP), profilin (3-1E), and lactate dehydrogenase have been studied as anticoccidial subunit vaccine candidates (Blake et al., 2017; Olajide et al., 2022; Britez et al., 2023). These antigens have been demonstrated to have anticoccidial activity through challenge infection after vaccination in the form of a single antigen or a combination of multiple antigens with or without adjuvants. Recombinant and DNA vaccines, in particular, require subcutaneous or intramuscular administration to large numbers of chickens, making them difficult to use on farms. Therefore, their effectiveness is being tested in the laboratory or on a small scale. Their efficacy has generally been shown to range from 30% to 90% based on fecal oocyst numbers, lesion scores, and/or weight gains (Ahmad et al., 2016; Clark et al., 2017).

Although the results of studies using recombinant antigens and DNA vaccines are currently difficult to apply to a wide range of the poultry industry, many scientists are continuing to study the anticoccidial effects of antigens for the future. The ROP21 protein secreted by *E. tenella* was produced in prokaryotic cells and administered subcutaneously as a recombinant antigen to chickens, which were then challenged with homologous *Eimeria* species. Immunized chickens exhibited increased secretion of IFN-γ and IL-4, along with increased weight gain, reduced lesion scores and decreased fecal oocyst shedding (Shi et al., 2023). Chickens were intramuscularly vaccinated with recombinant elongation factor 2 (EF2), an immunogenic common antigen across *Eimeria* species, and then challenged with *E. acervulina*, *E. maxima* and *E. tenella*, respectively. The vaccinated chickens had decreased body weight loss, oocyst outputs and intestinal lesion scores, and elevated levels of IFN-γ and IL-4 mRNA levels (Mi et al., 2023). Chickens were subcutaneously inoculated with the AMA1 antigen of *E. tenella*, which is expressed in sporulated oocysts and sporozoites, not in merozoites, and then challenged with the same *Eimeria* species. AMA1 vaccination induced the reduction of oocyst production, whereas no significant differences in oocyst production were found in chickens vaccinated with AMA2, which is expressed only in merozoites (Pastor-Fernández et al., 2018).

Instead of recombinant antigens, inactivated *E. tenella* whole-sporozoites emulsified in nanoparticle adjuvant were subcutaneously inoculated into chickens, followed by challenge with the same strain and the inactivated sporozoite vaccine protective efficacy was evaluated using the anticoccidial index (ACI), a composite metric incorporating survival rate, relative weight gain, lesion scores, and oocyst shedding (Juárez-Estrada et al., 2021a). Computed ACI values are interpreted as excellent protective efficacy (ACI>180), moderate (ACI: 160–179), limited (ACI: 120–159), or no protective efficacy (ACI< 120) (Zhao et al., 2019). Based on an anticoccidial index of 186, sporozoite-vaccinated chickens were effectively protected against challenge infection (Juárez-Estrada et al., 2021a). Similarly, a DNA vaccine containing one AMA3 epitope and two RON2 (rhoptry neck protein 2) epitopes of *E. tenella* was injected intramuscularly into chickens and then challenged with the homologous strain. DNA-vaccinated chickens had lower lesion scores and fecal oocyst counts, and higher transcript levels of IL-2, IL-10, and IFN-γ (Chen et al., 2024). A DNA vaccine containing *E. tenella* rhoptry protein 27 (ROP27) showed anticoccidial effects against *E. tenella* challenge, including increased weight gain, reduced lesion scores, and decreased oocyst excretion. It also increased the transcript levels of IL-2, IL-6, and IFN-γ, but not IL-4 and IL-10 (Zhao et al., 2025). Interestingly, chickens immunized with the ROP27 recombinant protein showed similar anticoccidial protection against *E. tenella* infection (Li et al., 2023). A DNA vaccine encoding four common antigens conserved among *Eimeria* species (14-3-3, elongation factor 2, glyceraldehyde-3-phosphate dehydrogenase, and transhydrogenase) was intramuscularly injected into chickens, which were then challenged with three different *Eimeria* species. The spleens of DNA-vaccinated chickens showed increased CD4^+^ and CD8^+^ T cells, serum IgG antibody levels, and transcript levels of IL-2, IL-4, and IFN-γ. Vaccination of chickens has been shown to reduce intestinal lesions, alleviate weight loss, and reduce oocyst production (Chen et al., 2023). Additionally, self-replicating live bacterial vectors, such as *Mycobacterium*, *Saccharomyces*, *Bacillus*, *Salmonella*, and *Lactobacillus*, have been used to express *Eimeria* antigens and deliver them orally to protect against *Eimeria* infection. Chickens immunized with *Lactobacillus plantarum* expressing the TA4-AMA1 and profilin antigens of *E. tenella* had increased levels of CD4^+^ and CD8^+^ T cells, IgA levels, or transcript levels of IL-2 and IFN-γ. Vaccination has been shown to reduce intestinal lesions, alleviate weight loss, and decrease oocyst production in chickens infected with *E. tenella* (Liu et al., 2021; Pan et al., 2023). Similarly, birds immunized orally with *Lactococcus lactis* expressing the immune mapped protein1 (IMP1) antigen of *E. tenella* showed anticoccidial effects against homologous *Eimeria* challenge (Ma et al., 2021). In general, vaccines using single antigens or recombinants of multiple antigens have been shown to be partially effective in reducing oocyst counts, intestinal lesion scores, and/or body weight loss, suggesting that there are still limitations to using antigen-based vaccines as a substitute for live vaccines, considering the effectiveness of the recombinant vaccines and the practical difficulties of administering the recombinant vaccines in the field.

#### 6.3.2 Non-attenuated and attenuated vaccines

Generally, non-attenuated and attenuated vaccines are produced by mixing sporulated oocysts of at least three different *Eimeria* species and are delivered to the host by drinking water, spraying, or inoculating embryonated eggs. Protective immunity can generally be developed when chicks are infected with a single high dose or multiple low doses of the *Eimeria* parasite. Commercial anticoccidial vaccines, which induce the formation of protective immunity after multiple low-dose infections, induce protective immunity in the host through two to three fecal-oral infection cycles, and have become a successful strategy for controlling and preventing coccidiosis outbreaks in the poultry industry (Peek and Landman, 2011; Ahmad et al., 2016; Jenkins et al., 2023; Liu et al., 2023). Non-attenuated vaccines contain *Eimeria* strains that have been isolated in the field or maintained in the laboratory without alteration in virulence and pathogenicity. Attenuated vaccines contain strains of *Eimeria* that have been modified to reduce virulence and pathogenicity by either continuous passages of the parasite through embryonated eggs or by selecting precocious oocysts. Precocious parasites are characterized by a shorter intestinal life cycle, which reduces the total time required for the two to three fecal-oral infection cycles required for protective immunity (Peek and Landman, 2011; Ahmad et al., 2016). Unlike non-attenuated vaccines, the production of attenuated vaccines requires more complicated steps, which increases production costs and makes it difficult to induce attenuation of virulence while maintaining immunogenicity for effective protective immunity (Ahmad et al., 2016; Liu et al., 2023).

The important point is that a single life cycle after vaccination with low-dose sporulated oocysts of several *Eimeria* species is unlikely to confer complete protective immunity against high-dose infection. Therefore, this typically requires two or three reinfections through the fecal-oral transmission cycle of the vaccine parasites in existing the litter or bedding over a period of 2–4 weeks after vaccination (Peek and Landman, 2011; Blake et al., 2017). Other concerns may be the risk of reversal of virulence of live vaccines and increased gut stress depending on farm conditions (Liu et al., 2023). In addition, although the impact on the emergence and extent of genetic diversity at the level of the *Eimeria* genome has not yet been determined, analysis of several genes encoding recombinant vaccine candidate proteins suggests that host immunity does not contribute significantly to genetic selection (Blake et al., 2017; Clark et al., 2017). Although *E. maxima* is somewhat of an exception, this suggests that *Eimeria* vaccines have a very high probability of protecting against field infections, although testing with a wider range of isolates is needed. On the other hand, live vaccine use can reduce or eliminate public health concerns by reducing the use of antibiotics, as it increases the proportion of *Eimeria* parasites that are not resistant to anticoccidial drugs. There are very few reports on large-scale field trials of live vaccines. When a commercial coccidia vaccine was sprayed onto 1-day-old chicks, productivity increased compared to past farm history, but oocyst shedding patterns and intestinal lesion scores varied greatly depending on farm environment conditions (Nguyen et al., 2024). Although studies on large-scale field trials of live vaccines are limited, live vaccines have been shown to be effective in preventing coccidiosis under field conditions. However, the effectiveness of live vaccines can be influenced by various factors, including farm temperature, litter condition, humidity, farm management, use of anticoccidial agents, immunosuppressive diseases, the composition of the gut microbiota, or chicken density. Therefore, additional field trials are needed to provide more definitive data. Furthermore, the assessment of these vaccination programs benefits from multi-flock evaluations, typically over two or three successive cycles, rather than a single flock analysis, as field outcomes may differ once the system reaches a new equilibrium of oocyst cycling and flock immunity.

Recently, genetic manipulation of *Eimeria* parasites by transgenic technique and gene editing has been used for several *Eimeria* species, opening up the possibility of influencing or modulating the immunogenicity, virulence, or life cycle duration of *Eimeria* species (Pastor-Fernández et al., 2018; Fatoba and Adeleke, 2020; Liu et al., 2023). Vaccination of transgenic *E. tenella* expressing AMA1 of *E. maxima* protected against *E. maxima* challenge with a significant reduction of oocyst output (Pastor-Fernández et al., 2018). Furthermore, vaccination of chickens with a mixture of transgenic *E. tenella* expressing either AMA1 or IMP1 (immune mapping protein-1) of *E. maxima* reduced fecal cyst shedding, lesion scores and weight loss induced by *E. maxima* challenge (Pastor-Fernández et al., 2020). Gene editing using the clustered regularly interspaced short palindromic repeats (CRISPR)/Cas9 (endonuclease) system was achieved by inserting a red florescent gene into the c-terminal of the MIC2 gene of *E. tenella* (Tang et al., 2020). Genome editing targeting histone H4 in *E. tenella* was developed using FnCas12a protein-crRNA, which belongs to ribonucleoprotein (RNP)-mediated gene editing (Cheng et al., 2021). Thus, transgenic *Eimeria* and genome editing could be useful tools for a vaccine delivery system and could potentially be developed as a pillar of chicken coccidia vaccination strategies.

### 6.4 Natural and biological approaches to coccidiosis control

**Figure 1:**
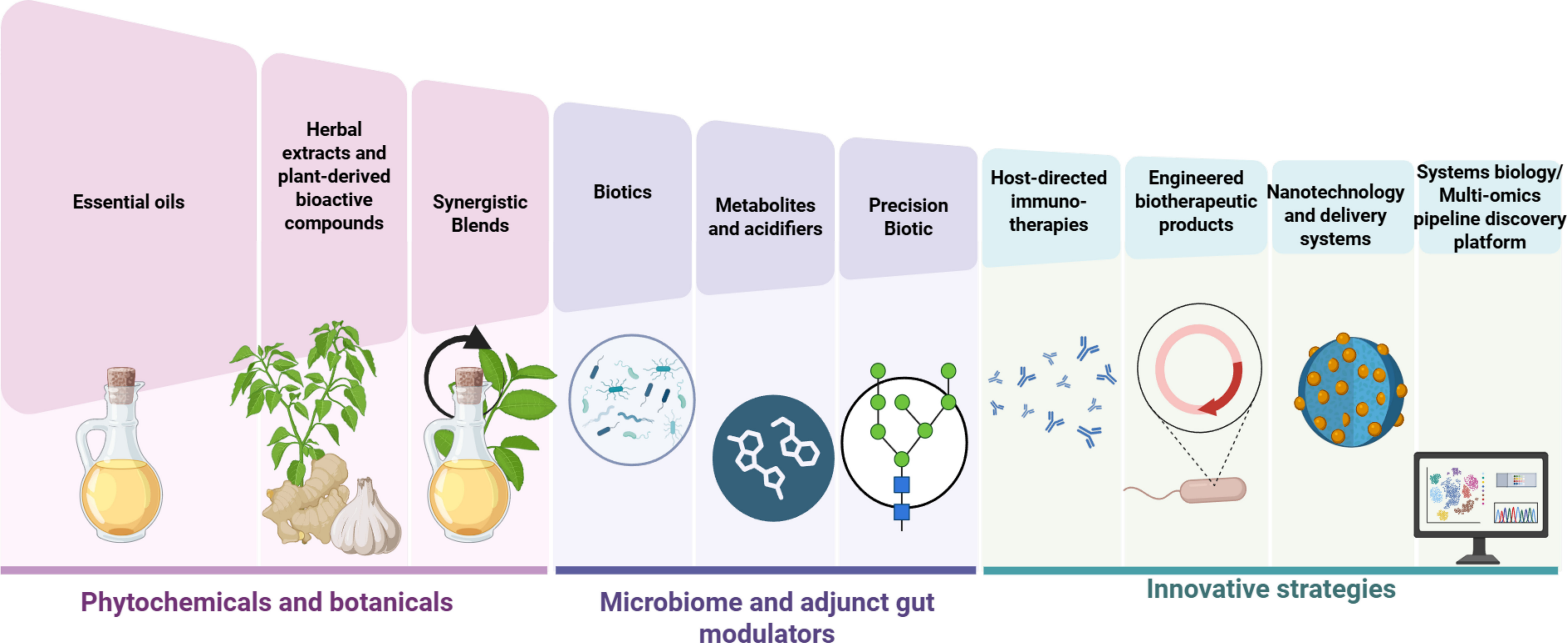
Conceptual framework of antibiotic-free interventions for *Eimeria* in poultry. A schematic representation summarizing the major non-antibiotic established strategies (phytochemicals and botanical), expanding strategies (microbiome and adjunct gut modulators) and emerging omics-guided innovations (Innovative strategies) for sustainable coccidiosis control in the poultry industry. Image was created with BioRender.com.

Decades of long-term reliance on ionophores and synthetic coccidiostats have practically controlled *Eimeria* infection in intensive poultry production, leading to the emergence of drug-resistant strains, and ultimately leading to increased regulation on the use of anticoccidials in livestock production, and consumer demands for antibiotic-free production (Flores et al., 2022; Martins et al., 2022; Zhou et al., 2025). As a result, research efforts have shifted to non-pharmacological and more sustainable strategies that not only inhibit parasite development but also enhance intestinal barrier integrity, regulate host immune responses, and restore gut microbial homeostasis, which collectively increase the resistance of chickens to *Eimeria* infection (Ahmad et al., 2024). These efforts are broadly categorized into three complementary domains: (i) phytochemicals and botanicals; (ii) microbiome-based and gut modulators; and (iii) innovative immunologicals and omics-guided interventions (**Figure 1**). A comparative summary of the natural biological strategies for managing *Eimeria* spp. infection in this current paper is presented in **Table 7**, whereas more exhaustive insights and a detailed review of non-antibiotic control strategies in chicken coccidiosis are provided elsewhere (Flores et al., 2026).

**Table 7:** Summary table of natural and biological approaches for *Eimeria* spp. infection.

Category	Components/Examples	Primary mechanisms	Key outcomes	Reference
Phytochemicals and botanicals	Essential oils (carvacrol, thymol, eugenol), allicin, artemisinin and its derivatives, berberine, plant derived metabolites and extracts (flavonoids, tannins, saponins, complex polysaccharides)	membrane disruption or sporulation inhibition, host- immunomodulation, anti-inflammatory and antioxidant activity, gut modulation through intestinal barrier integrity modulation or gut microbial modulation	Inhibition of sporulation and sporozoite invasion, impair oocyst wall, improved FCR and weight gain in birds, reduced oocyst shedding and lesion score, intestinal barrier protection	Wang et al., 2008; Lee et al., 2010b; Fatemi et al., 2015; Jiao et al., 2018; Chang et al., 2021; Abd-Elrahman et al., 2022; Coroian et al., 2022; Han et al., 2022; Zhang et al., 2023a; El-Ghareeb et al., 2023; Ristanti et al., 2024; Tchodo et al., 2024; Wang et al., 2024b; Nguyen et al., 2025
Microbiome-based and gut-modulating	Prebiotics (MOS, inulin, FOS, β-glucans from yeast or cell walls), probiotics (*Lactobacillus*, *Bifidobacterium*, *Bacillus*, *Saccharomyces)*, synbiotics, postbiotics, microbial metabolites, precision biotics	Competitive exclusion, SCFA production, restoration of microbial homeostasis	Improve intestinal villus architecture, improve mucosal immunity, improve oxidative balance, improve weight gain, reduced lesion score, reduced oocyst shedding	Gómez-Verduzco et al., 2009; Cox et al., 2010; Ghasemi et al., 2010; Bortoluzzi et al., 2017; Leung et al., 2019a, b; Gomez-Osorio et al., 2021; Blokker et al., 2022; Gomez Quintero et al., 2022; Mohsin et al., 2022; Leone and Ferrante, 2023; Liu et al., 2024; Ogwiji et al., 2024; Dong et al., 2025; Kolli et al., 2025; Sumon et al., 2025
Immunonologicals	HDPs (NK-lysin, β-defensin-1), hyperimmune egg yolk antibodies (IgY), recombinant vaccines (EtMIF, EF-1α) and transgenic vaccines	Immunomodulatory, targeted cytotoxicity to *Eimeria* spp. sporozoites	Passive protection, downregulation of pro-inflammatory cytokines	Lee et al., 2013; Kim et al., 2021; León-Núñez et al., 2022; Lee et al., 2023; Mahmoud et al., 2023; Bai et al., 2025
Advanced tools	Engineered biotherapeutics (live vectors harboring Eimeria antigens), nanovaccines, CRISPR/Cas9, omics-guided vaccine design	Delivery of *Eimeria* antigens via microbial or protozoan vectors, precise multi-epitope targeting improved bio-kinetics of natural cargos	Durable cellular immunity, genome-edited delivery systems, cross-species protection	Jenkins et al., 2018; Partovi et al., 2019; Pastor-Fernández et al., 2020; Mohsin et al., 2021; Soutter et al., 2022; Yu et al., 2022; Xu et al., 2022; Youssefi et al., 2023; Algammal et al., 2024; Sanchez-Arsuaga et al., 2025

EF-1α, *Eimeria* elongation factor-1α; EtMIF, *Eimeria tenella* macrophage migration inhibitory factor; FOS, fructooligosaccharides; HDP, host defense peptide; MOS, mannan-oligosaccharides.

#### 6.4.1 Phytochemicals and botanicals

Phytochemicals and botanicals represent a diverse group of bioactive compounds and extracts with well-established antimicrobial parasite-directed interference (i.e., membrane disruption or sporulation inhibition) and host-mediated benefits, including immunomodulation, anti-inflammatory and antioxidant activity, and gut modulation through intestinal barrier integrity modulation or gut microbial modulation (Ristanti et al., 2024; Wang et al., 2024b). Essential oils (EOs) are volatile aromatic extracts from plants that frequently combine direct anticoccidial effects with mucosal protection. EOs of oregano (*Origanum vulgare*), thyme (*Thymus vulgaris*), and clove (*Syzygium aromaticum*), rich in carvacrol, thymol and eugenol, have been consistently reported to reduce fecal oocyst shedding and intestinal lesion score, while decreasing the feed conversion rate (FCR) and improving weight gain in *Eimeria* infection (Mohiti-Asli and Ghanaatparast-Rashti, 2015; Zhang et al., 2023a). These oils have also demonstrated direct antiparasitic potential by damaging oocysts membranes and reducing sporulation rates (Remmal et al., 2011; Ristanti et al., 2024). A previous report found that oregano oil increased the expression of tight-junction proteins (ZO-1, Claudin-1) and improved intestinal morphology during mixed *Eimeria* infection, indicating modulation of the intestinal barrier (Zhang et al., 2023a). Another EO constituent, cinnamaldehyde from cinnamon (*Cinnamomum* spp.), can mitigate *Eimeria-*induced damage while increasing cell proliferation in chicken spleen lymphocytes, antibody responses to EtMIC2, nitric oxide production in macrophages and expression of cytokines IL-1β, IL-6, IL-15 and IFN-γ in the intestine of chickens, demonstrating combined antiparasitic and immunomodulatory effects (Lee et al., 2010b; Orengo et al., 2012; Qaid et al., 2021).

Beyond volatile EOs, non-EOs phytometabolites also exhibit potent anticoccidial activity through both parasite-directed and host-mediated mechanisms. Allicin from *Allium sativum* exerts a direct parasitic effect on *Eimeria* oocysts, alleviates intestinal lesions and oocyst production, while enhancing systemic and mucosal antibody responses in *E. tenella* (Chang et al., 2021; Abd-Elrahman et al., 2022). Artemisinin and its derivatives from *Artemisia* spp. were reported to inhibit the sporulation of oocysts and sporozoite invasion *in vitro*, and to impair the oocysts wall, while suppressing NF-κB and IL-17A-mediated inflammation, modulating gut microflora and improving intestinal barrier functions in supplemented infected birds (Fatemi et al., 2015; Jiao et al., 2018; Coroian et al., 2022; Han et al., 2022). Similarly, berberine, an isoquinolone alkaloid derived from *Coptis chinensis* and *Berberis* spp., has demonstrated both antiprotozoal and host-protective effects by reducing oocyst outputs and lesion scores for *Eimeria* spp., upregulating genes associated with epithelial integrity, and attenuating oxidative and inflammatory stress (Zhu et al., 2019; Nguyen et al., 2021, 2025).

Complementary plant-derived metabolites and extracts, including flavonoids, tannins, saponins, and complex polysaccharides, also confer a well-documented benefit against avian coccidiosis through a multi-target mode of action involving direct parasiticidal activity such as inhibition of oocyst sporulation and interference with sporozoite invasion or intracellular development, antioxidant support, and host immunomodulation (Flores et al., 2026). For instance, neem (*Azadirachta indica*) limonoid and flavonoid extracts showed direct parasite interference in addition to improving performance indices in chickens and mitigating cecal pathology, thereby lowering oocyst shedding and mortality in *Eimeria*-infected birds (Tipu et al., 2002; Biu et al., 2006; Tchodo et al., 2024). Additionally, phenolics/flavonoid-rich extracts from *Bidens Pilosa* can act at different developmental stages of the parasite, reinforce intestinal barrier integrity, and aid microbial shifts to beneficial taxa in the gut (Chang et al., 2016; Yang et al., 2019; Memon et al., 2021). Saponins from *Yucca schidigera* and *Quillaja Saponaria* were found to improve gut morphometry and exert immunostimulatory effects to consistently reduce weight loss, FCR, and lesion outcomes with known synergistic activity alongside vaccination or low-dose ionophores for additional protection against *Eimeria* challenge (Oelschlager et al., 2019; Bafundo et al., 2020, 2021). Tannins, particularly tannic acid and proanthocyanidins, reduced oxidative stress and intestinal tissue damage, thereby promoting faster mucosal recovery and reduced lesion scores (Wang et al., 2008).

The flavonoids myricetin and quercetin are reported to inhibit lipid peroxidation and expression of inflammatory mediators (IL-1β, IL-6, TNF-α, CCL20, CXCL13, and avian defensins AvBD16), leading to reduced intestinal damages and oocyst production, and improved growth performance (El-Ghareeb et al., 2023). Similarly, complex polysaccharides (β-glucans and heteropolysaccharides from mushrooms and medicinal herbs) and lectins also augment anticoccidial effects by activating macrophages and lymphocytes, modulating nitric oxide production and cytokine production, and enhancing weight gain and immune resilience during *Eimeria* infection (Dalloul et al., 2006; Lee et al., 2010b). Collectively, these phytochemicals and botanicals act through complementary pathways by directly targeting *Eimeria* development while modulating oxidative balance, immune signaling, and microbiota composition to alleviate *Eimeria-*induced pathogenesis and accelerate recovery. Despite the multitarget mechanisms of these botanical formulations, future work should prioritize chemical characterization, standardized formulations, and large-scale validation to realize their full potential as sustainable and residue-free anticoccidial solutions.

#### 6.4.2 Microbiome-based and gut-modulating approaches

The chicken gastrointestinal tract harbors a complex microbial ecosystem that is essential for nutrient absorption, immune maturation, and pathogen exclusion (Clavijo and Flórez, 2018). *Eimeria* infection disrupts this equilibrium, and thus modulating the gut microbiota and intestinal milieu has emerged as a promising complementary strategy to conventional anticoccidials. These approaches include prebiotics, probiotics, synbiotics, postbiotics, microbial metabolites, and precision biotics, each targeting the restoration of microbiota, barrier protection, and immune regulation to enhance host resilience against *Eimeria* challenge. Prebiotics, such as mannan-oligosaccharides (MOS), inulin or fructooligosaccharides (FOS) and β-glucans (yeast or fungal cell walls), are non-digestible feed ingredients that nurture the growth and activity of bacteria already present in the gut (Teng and Kim, 2018). In coccidia challenge, these prebiotics improved the villus architecture, mucosal immunity and oxidative balance while also lowering fecal oocyst output and lesion score in supplemented infected birds (Gómez-Verduzco et al., 2009; Cox et al., 2010; Leung et al., 2019b). Specifically, MOS enhances feed efficacy, mucosal IgA, and immune organ development, whereas yeast- and sugarcane-derived prebiotics improve intestinal pH, short-chain fatty acid profiles (SCFA), and weight gain (Gómez-Verduzco et al., 2009; Leung et al., 2019a, b; Ogwiji et al., 2024).

Probiotics are live, nonpathogenic microorganisms such as *Lactobacillus*, *Bifidobacterium*, *Bacillus*, and *Saccharomyces*, that can benefit the host by competitively excluding pathogens, improving intestinal barrier function, immunomodulation, and neurotransmitter synthesis when administered in adequate amounts (Latif et al., 2023). Supplementation with *Lactobacillus plantarum* alleviated coccidia-related adverse effects, and supplementation with *Saccharomyces cerevisiae* provided additional protection by reinforcing gut barrier integrity and enhancing both cell-mediated and humoral immune signaling in broilers (Mohsin et al., 2022; Ogwiji et al., 2024). Another microbiome-based approach is synbiotics, which is the combination of probiotics and prebiotics in different proportions to confer benefits on the host (Gomez Quintero et al., 2022). Evidently, synbiotic formulations combining probiotic strains such as *Enterococcus faecium*, *Lactobacillus casei*, *L. plantarum*, and *Pedicoccus acidilactici* with complex matrix of prebiotics and additives, including inulin, oligofructose, FOS, various carbohydrates like dextrose and maltodextrin, bacterial cell wall fragments and phycophytic substances significantly improve growth parameters. These formulations further contributed to the modulation of oxidative markers, reduced oocyst production, and lower lesion score during coccidia infection (Ghasemi et al., 2010; Ogwiji et al., 2024).

Emerging evidence also highlights the beneficial effects of postbiotics, or preparations of inanimate microorganisms and/or their components, in conferring benefits to the host (Vinderola et al., 2023). In a recent necrotic enteritis infection closely associated with *Eimeria* infection, postbiotic supplementation significantly improved intestinal villi and crypt structures and increased the expression of mucin-2, olfactomedin-4, and zonula occludens-1, while reducing inflammatory cytokines in infected birds (Dong et al., 2025). Adjunct gut modulators such as microbial metabolites (short chain fatty acids and medium chain fatty acids) and novel precision biotics which are engineered glycans act through indirect host-mediated mechanisms to alleviate the consequences of coccidiosis and increase overall resistance to this parasitic infection by acting as signal molecules, regulatory agents or substrates to steer gut microbial metabolism and pathways to maintain gut homeostasis (Bortoluzzi et al., 2017; Gomez-Osorio et al., 2021; Blokker et al., 2022; Leone and Ferrante, 2023; Liu et al., 2024; Kolli et al., 2025; Sumon et al., 2025). Collectively, these microbiome- and gut-oriented interventions alleviate the clinical consequences of coccidiosis by restoring intestinal homeostasis and enhancing host defense mechanisms. However, key challenges remain, including the variability in microbial strains and formulations, inconsistent dosing and delivery regimens, limited validation, and the lack of information on harmonized efficacy endpoints to enable scalable implementation.

#### 6.4.3 Innovative immunologicals and omics-guided interventions

A new generation of immunological, molecular, and nanotechnological tools is helping to mitigate coccidiosis with the targeted modulation of host-parasite interactions and controlled delivery of protective biologics. To date, these strategies include host-directed immunotherapies, engineered biotherapeutics, nanotechnology-based delivery systems, and omics-guided interventions, all designed to elicit durable mucosal immunity, limit parasite replication, and reduce reliance on conventional anticoccidials. Host-directed immunotherapies, such as host defense peptides (HDPs), hyperimmune egg yolk antibodies (IgY), recombinant vaccines, and transgenic vaccines, are emerging frontiers in poultry health, specifically designed to elicit defined immune protection. Avian HDPs, such as NK-lysin, have shown combined immunomodulatory effects, and directed cytotoxicity against various *Eimeria* spp. sporozoites *in vitro* (Lee et al., 2013; Kim et al., 2021). Chicken β-defensin-1 and NK-lysin exhibited bonafide anticoccidial activity in *Eimeria*-challenged broilers *in vivo* (Lee et al., 2013; Mahmoud et al., 2023). Immunoglobulin Y (IgY) is a functional equivalent of mammalian IgG in birds, reptiles, and amphibians, and is the primary humoral immune defense molecule in avian species (León-Núñez et al., 2022). Hyperimmune egg yolk antibodies (IgY) produced from hens immunized with *Eimeria* antigens confer passive protection by blocking epithelial invasion and reducing lesion score, fecal oocyst shedding and weight loss induced by *Eimeria* infection (Lee et al., 2009b, 2010a; Juárez-Estrada et al., 2021b). Contemporary advanced host-directed immunotherapeutics with *Eimeria*-directed antigens such as recombinant EtMIF (macrophage migration inhibitory factor) or EF-1α (*Eimeria* elongation factor-1α) in *Eimeria*-challenged birds also alleviated coccidiosis with improved mucosal immunity, downregulation of inflammatory cytokines, and reinforcement of epithelial tight junctions (Lee et al., 2023; Bai et al., 2025).

Another advanced class of biologics includes engineered biotherapeutics that use live microbial or protozoan vectors to deliver immunoprotective antigens or immunomodulators. Although it is a growing field, several studies have already reported the use of live vectors such as *Eimeria* or *Saccharomyces cerevisiae* to deliver immunogenic *Eimeria* antigens (apical membrane antigens (AMAs), immune mapped protein-1 (IMP-1), microneme protein 3 (MIC3) to confer protective immunity against this parasitic disease while improving growth performance and mitigating coccidia-induced pathologies (Pastor-Fernández et al., 2020; Soutter et al., 2022). Beyond recombinant technology, modern drug delivery platforms using nanotechnology are also currently being explored for parasite control to improve bioavailability, epithelial residence, and kinetics of natural products and recombinant biologics (Xu et al., 2022; Tiwari et al., 2023). In a recently reported coccidiosis model, the nanoemulsion of eugenol and essential oils of clove, as well as the nanoencapsulation of curcumin and *Azadirachta indica* leaf extract, enhanced antioxidant defense of the cargos. Supplementation also reduced weight loss in chickens infected with *Eimeria* spp (Partovi et al., 2019; Youssefi et al., 2023; Algammal et al., 2024). Similarly, recombinant nanovaccines encapsulating *Eimeria* antigens were also able to prompt strong humoral and cellular immunity in challenged chickens in addition to lower mortality and lesion score (Jenkins et al., 2018; Xu et al., 2022). Omics-guided discovery platforms now extend these efforts by mapping host-parasite-microbiome interactions to identify vaccine antigens and metabolic targets to enable multi-epitope and genome-edited designs to improve cross-species protection (Mohsin et al., 2021; Yu et al., 2022; Sanchez-Arsuaga et al., 2025). Together, these innovative platforms integrate immunological precision and technological delivery to achieve multilevel protection against coccidiosis. For broader application, they require standardized formulations, validated dosing, scalable manufacturing, and long-term field trials to confirm safety, efficacy, and cost-effectiveness for commercial deployment.

## 7 Conclusions and future perspectives

The poultry industry has long been an important part of the human diet, providing chickens and eggs, and has always provided readily available, high-quality protein. In particular, the industry continues to suffer economic losses due to infection of *Eimeria* species, among many other diseases. To reduce economic losses caused by coccidiosis, anticoccidials have been widely used as feed additives or control methods in commercial and domestic chickens, but this has led to problems with drug residues and the emergence of drug-resistant strains (Martins et al., 2023). A variety of alternative control strategies, including vaccines and natural and biological approaches, are being implemented in poultry farms, and their effectiveness is gradually being proven. Therefore, efforts are being made to secure highly effective substances, and various methods to increase their effectiveness are being studied (Lee et al., 2022; Wickramasuriya et al., 2022; Zaheer et al., 2022; Li et al., 2025a, b). A comprehensive and in-depth understanding of coccidiosis, including the *Eimeria* life cycle, protective immunity and control strategies, is vital for a profitable poultry industry, which is facing new global challenges. Continuously updated information on coccidiosis will provide a solid understanding of how to better control coccidiosis in the field. As technology advances and our understanding of the complexities of protective immunity against *Eimeria* species improves, significant advances in the management of coccidiosis are expected. This will lead to more effective and cost-effective disease prevention and treatment.

**Figure 2:**
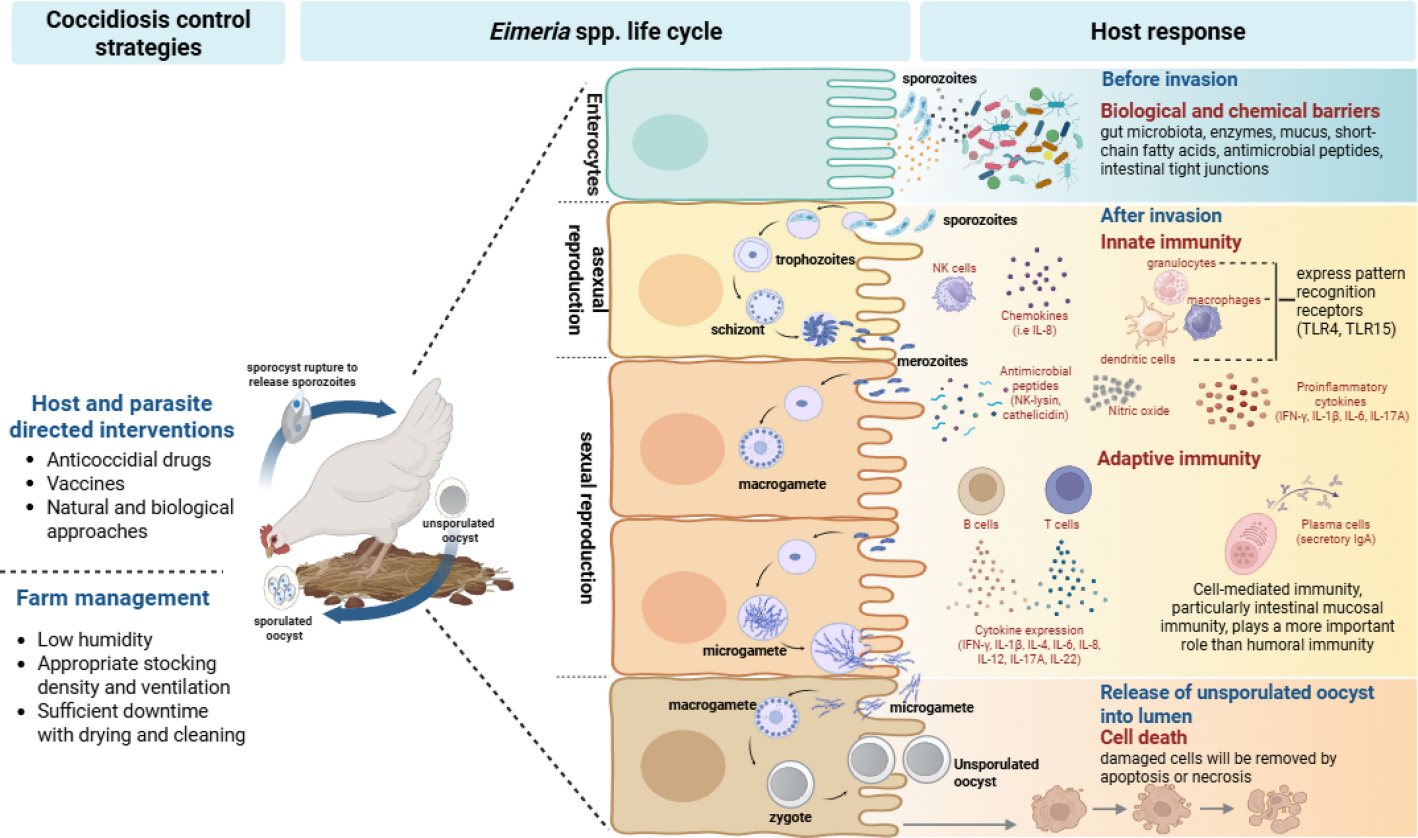
Integrative landscape of *Eimeria* spp. life cycle, host immunity and multifaceted control intervention points for coccidiosis control. The schematic illustrates the exogenous and endogenous phases of *Eimeria* spp. life cycle in relation to host parasite dynamics. Under optimal environmental conditions (i.e. temperature and humidity), unsporulated oocyst in the litter or environment undergo sporulation and become infective. Upon oral ingestion of sporulated oocyst, sporulated oocyst undergo mechanical and enzymatic rupture within the digestive tract, releasing sporozoites. These sporozoites invades intestinal epithelium, initiating successive rounds of schizogony then gametogony, ultimately resulting to the development of zygote which is excreted in the feces as unsporulated oocysts. During the initiation of parasite invasion, host biological and chemical barriers serves as primary defense mechanism. Once invasion commences, several defense strategies of the innate and adaptive immunity are activated in the host’s intestines. Key control strategies are mapped onto the corresponding stages of the parasite life cycle, integrating farm management practices that reduce oocyst survival, parasite directed approaches and host directed interventions to mitigate transmission and pathogenesis. IL, interleukin; IFN-γ, interferon gamma; TLR, Toll-like receptor. Image was created with BioRender.com.

Reducing economic losses from coccidiosis requires an integrated management strategy based on vaccination, good farm management, use of probiotics/prebiotics, and understanding of coccidiosis (**Figure 2**). Among these, genetically modified vaccines will play a key role in addressing concerns about the emergence of drug-resistant strains and drug residues in meat. Transgenic *Eimeria* strains introducing PAMPs or immunogenic molecules using the CRISPR/Cas9 system or gene editing are expected to enhance innate and adaptive immunity. Since the frequently occurring *Eimeria* species vary depending on the regions or production systems, commercial coccidia vaccines are produced by mixing about 3–5 *Eimeria* species. Therefore, selecting a vaccine that is appropriate for the situation through periodic survey will help reduce the damage caused by coccidiosis.
